# FGF1-FGFR2 axis regulated by nuclear receptor RORγ represents an effective strategy in intrahepatic cholangiocarcinoma

**DOI:** 10.1038/s41420-025-02844-8

**Published:** 2025-12-22

**Authors:** Zhanfeng Gu, Xiaojuan Wang, Hong Wang, Junhua Wang, Zhaorong Huang, Dongyue Pan, Zhenhua Zhang, Yechun Zeng, Guodi Cai, Huizi Sun, Jun Zheng, Yichu Nie, Qingwen Zhang, Haolong Li, Franky Leung Chan, Junjian Wang, Jianwei Zheng, Yingfang Fan

**Affiliations:** 1 Department of Hepatobiliary surgery, The Third Affiliated Hospital, Southern Medical University, Guangdong, 510630 Guangzhou China; 2 Hepato-Pancreato-Biliary Center, Beijing Tsinghua Changgung Hospital, Key Laboratory of Digital Intelligence Hepatology (Ministry of Education), School of Clinical Medicine, Tsinghua Medicine, Tsinghua University, 102218 Beijing, China; 3 National-Local Joint Engineering Laboratory of Druggability and New Drugs Evaluation, School of Pharmaceutical Sciences, Sun Yat-sen University, Guangdong, 510006 Guangzhou China; 4 Clinical Research Institute, The First People’s Hospital of Foshan, Guangdong, 528000 Foshan China; 5 The Breast Center, Cancer Hospital of Shantou University Medical College, Guangdong, 515041 Shantou China; 6 State Key Laboratory of Quality Research in Chinese Medicine and Institute of Chinese Medical Sciences, University of Macau, Taipa, Macao; 7 School of Biomedical Sciences, The Chinese University of Hong Kong, Sha Tin, Hong Kong

**Keywords:** Bile duct cancer, Oncogenes

## Abstract

Intrahepatic cholangiocarcinoma (iCCA) is a highly aggressive malignancy with limited therapeutic options. Although targeted therapies like pemigatinib provide partial clinical benefits, acquired resistance remains a significant challenge. Through integrative bioinformatics analysis of public datasets and immunohistochemical validation, we identified the retinoid-related orphan receptor gamma (RORγ) as markedly upregulated in iCCA. Genetic silencing and pharmacological inhibition of RORγ (GSK805/XY101) suppressed proliferation, induced apoptosis in vitro, and significantly reduced xenograft tumor growth in vivo. Mechanistically, RORγ promoted fibroblast growth factor receptor 2 (FGFR2) signaling via two complementary mechanisms: direct transcriptional activation of FGFR2 and induction of fibroblast growth factor 1 (FGF1) expression and secretion, which in turn activated FGFR2. Inhibition of RORγ markedly decreased FGF1 levels in conditioned media, whereas exogenous FGF1 restored tumor growth. Notably, RORγ antagonists synergized with pemigatinib to overcome resistance in pemigatinib-refractory models. Collectively, these findings identify the RORγ-FGF1-FGFR2 axis as a critical oncogenic driver in iCCA and highlight RORγ inhibition as a promising therapeutic strategy to suppress tumor progression and enhance sensitivity to FGFR inhibitors.

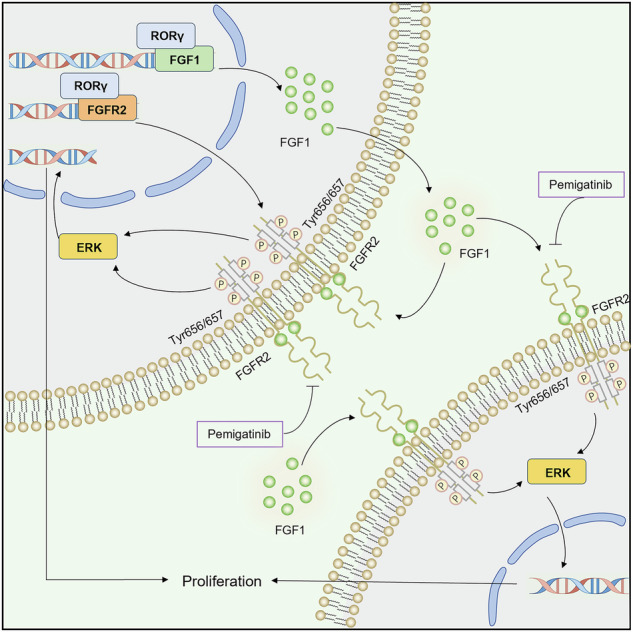

## Introduction

Cholangiocarcinoma (CCA) is a heterogeneous malignancy of the biliary tract, most frequently diagnosed at advanced stages and associated with poor prognosis [1, 2]. Among its subtypes, intrahepatic cholangiocarcinoma (iCCA) represents the second most common primary liver cancer and exhibits a uniquely rising global incidence compared with perihilar (pCCA) and distal (dCCA) subtypes [3–5]. Surgical resection remains the only potentially curative option, yet only a minority of patients are eligible, and recurrence rates remain high even after surgery [6]. iCCA is generally refractory to chemotherapy and radiotherapy, and the benefit of immunotherapy remains uncertain [7–9]. The approval of pemigatinib for FGFR2 fusion-positive iCCA marked an important step in precision oncology; however, its clinical impact is constrained by rapid development of acquired resistance and off-target toxicity [10]. These challenges underscore the insufficient understanding of iCCA progression and pemigatinib resistance [11, 12]. Defining the molecular pathways driving malignancy and drug resistance is therefore essential to identify new therapeutic targets, improve treatment efficacy, and ultimately overcome resistance in iCCA.

Nuclear receptors (NRs) are a large family of transcription factors that regulate diverse physiological processes, including cell growth, differentiation, metabolism, embryonic development, and organ function [13, 14]. Retinoid-related orphan receptor gamma (RORγ), encoded by *RORC*, together with retinoid-related orphan receptor A (RORα) and retinoid-related orphan receptor B (RORβ), forms a distinct subfamily of nuclear receptors [15, 16]. RORs exert their function by binding to specific genomic sites and regulating the transcription of genes involved in cell survival, apoptosis, metabolism, and immune regulation [17–19]. The RORγ gene encodes two isoforms: the thymus-specific RORγt and the ubiquitously expressed RORγ [20]. RORγt is indispensable for the differentiation of IL-17-producing T cells, which mediate both protective and pathogenic immune responses [21, 22]. Accordingly, targeting RORγt has emerged as a promising therapeutic approach for autoimmune diseases. Beyond immune regulation, accumulating evidence, including our own, has demonstrated that RORγ expression is elevated in multiple aggressive cancers, such as castration-resistant prostate cancer, hepatocellular carcinoma, osteosarcoma, pancreatic cancer, and triple-negative breast cancer, where it functions as an oncogenic driver [23–27]. Despite these advances, the role of RORγ in iCCA remains largely undefined and requires further investigation.

Fibroblast growth factor receptors (FGFRs) are transmembrane receptor tyrosine kinases (RTKs) that regulate key cellular processes, including proliferation, survival, and differentiation [28–30]. Their ligands, fibroblast growth factors (FGFs), bind to FGFRs and induce receptor dimerization, autophosphorylation, and subsequent activation of downstream signaling pathways [31, 32]. Dysregulated FGFR signaling has been strongly implicated in tumorigenesis, driving uncontrolled cell proliferation, migration, and metastasis [33]. Increasing evidence indicates that targeting the FGF-FGFR axis represents a promising therapeutic strategy.

Genetic and epigenetic modifications of FGFR2 result in its overexpression, fusion proteins, and enhanced ligand-binding affinity in tumor cells [29, 34]. FGFR2 fusions or rearrangements are particularly enriched in iCCA, accounting for 14% of cases [35]. In 2020, pemigatinib became the first FDA-approved targeted therapy for CCA patients with FGFR2 fusions or rearrangements [36, 37]. Despite initial efficacy, most patients rapidly develop acquired resistance, and the mechanisms driving resistance remain poorly understood [38, 39]. Furthermore, limited insights into the spatial and temporal regulation of FGFR2 activation in iCCA continue to hinder mechanistic understanding and the development of next-generation FGFR2-targeted therapies. Therefore, a more thorough understanding of the genomic and transcriptomic landscape of FGFR2 may facilitate the advancement of FGFR-targeted therapeutic strategies.

Here, our results identify RORγ as a novel therapeutic target and potential oncogenic driver in iCCA. Our study demonstrates that RORγ overexpression confers resistance to pemigatinib by upregulating FGF1 and FGFR2. iCCA cells secrete FGF1, which activates FGFR2 signaling to sustain tumor growth. Pharmacological inhibition of RORγ markedly suppresses the FGF1-FGFR2 signaling axis and reduces tumor growth and progression both in vitro and in vivo. Together, these findings establish that RORγ represents a promising therapeutic target for iCCA.

## Results

### RORγ is highly expressed in iCCA

**Fig. 1:**
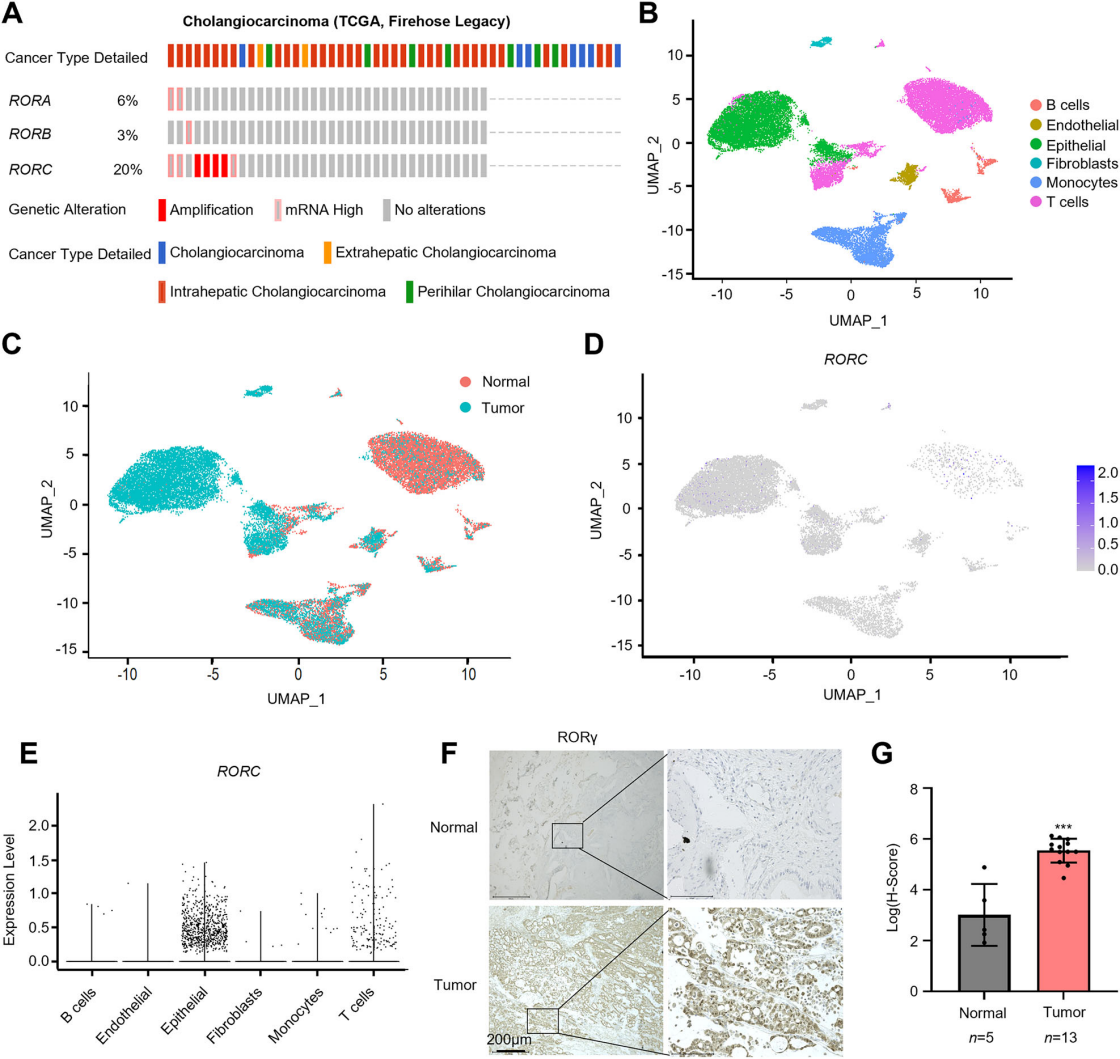
RORγ is highly expressed in iCCA. **A** Oncoprint display from cBioPortal of ROR family genes alterations in CCA tumors, with 51 queried patients/samples. **B**, **C** Single-cell sequencing analysis of iCCA patients (GSE138709) was conducted. Cells were grouped into distinct clusters based on their genomic profiles. Additionally, the cells were categorized by their origin, highlighting normal (N) and tumor (T) samples. **D**, **E** The mRNA levels of *RORC* across various cell types in tumor samples are presented. The plot illustrates *RORC* expression levels across different cell types, with notably higher expression observed in epithelial cells. **F**, **G** Representative images and analysis of IHC staining for RORγ expression in iCCA tumors (*n* = 13) and normal tissues (*n* = 5). Scale bars, 200 μm. Data are presented as mean ± SD. *p < 0.05, **p < 0.01, ***p < 0.001.

Nuclear receptors are known to play pivotal roles in the development of various cancers and are potential therapeutic targets. To investigate the function of RORγ in iCCA, we first examined genomic alterations of the ROR family using the cBioPortal platform. Our analysis revealed that *RORC* amplification and elevated mRNA levels were detected in 20% of cholangiocarcinoma cases, compared to other members of the ROR family, including *RORA* and *RORB* (Fig. 1A). To further explore the role of RORγ in iCCA, we conducted a transcriptome analysis utilizing published single-cell data (GSE138709). Cells were initially clustered based on their distinct genomic signatures, and *RORC* expression was analyzed. Our results indicated that single cells could be categorized into six subclusters (Fig. 1B, C), with tumor samples analysis revealing that *RORC* was predominantly expressed in epithelial cells (Fig. 1D, E). Furthermore, immunohistochemistry (IHC) was performed to evaluate RORγ expression in non-tumor gallbladder tissues and iCCA tissues. IHC analysis revealed significant upregulation of RORγ in iCCA tissues compared to non-tumor tissues (Fig. 1F, G). Taken together, these findings suggest that RORγ is overexpressed in iCCA and may play a critical role in tumor progression.

### RORγ inhibition suppresses iCCA survival both in vitro and in vivo

**Fig. 2:**
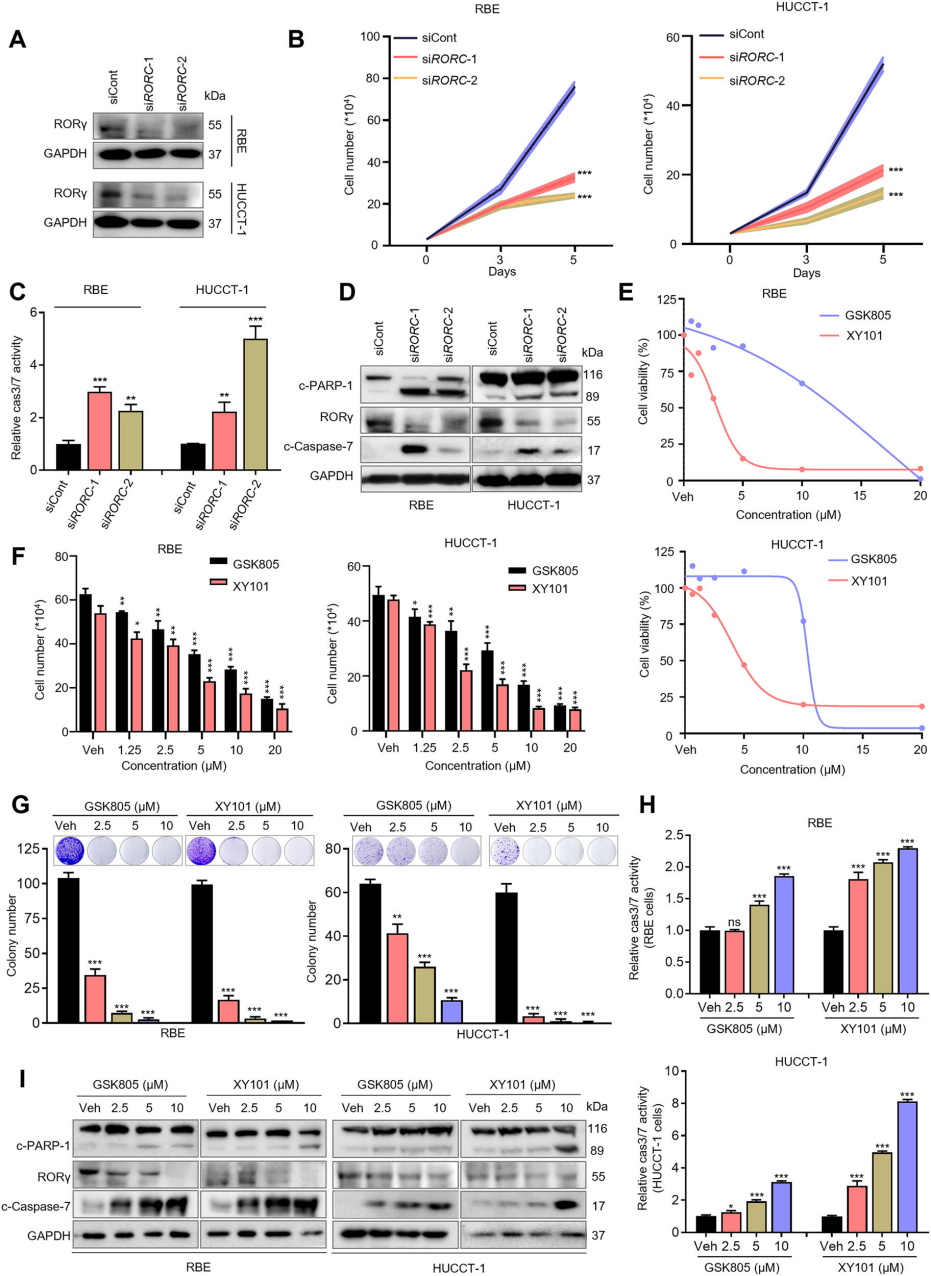
RORγ inhibition suppresses cell proliferation and induces apoptosis in iCCA. **A** Western blotting analysis was performed on RBE and HUCCT-1 cells transfected with *RORC* siRNAs or control. *n* = 3 biological replicates. **B** RBE and HUCCT-1 cells were transfected with control or *RORC* siRNAs, and live cells were counted at the indicated time points following transfection. *n* = 3 biological replicates. **C** RBE and HUCCT-1 cells were treated as in (**B**), and caspase-3/7 (cas3/7) activities were tested for apoptosis analysis. *n* = 3 biological replicates. **D** RBE and HUCCT-1 cells were transfected with *RORC* siRNAs or control, after which indicated proteins were detected by immunoblotting. *n* = 3 biological replicates. **E** An assay was conducted using Cell-Titer GLO (Promega) to assess the viability of RBE and HUCCT-1 cells treated with either a vehicle or specified concentrations of the RORγ antagonists GSK805 and XY101 over a period of 4 days. *n* = 3 biological replicates. **F** For RBE and HUCCT-1 cells that had been treated with the indicated concentrations of RORγ antagonists GSK805 and XY101 for 4 days, viable cells were counted following treatment. *n* = 3 biological replicates. **G** RBE and HUCCT-1 cells were treated with DMSO or the indicated concentrations of RORγ antagonists GSK805 and XY101 for 14 days, after which colony formation was measured. *n* = 3 biological replicates. **H** Caspase-3/7 (cas3/7) activities in RBE and HUCCT-1 cells were treated with the indicated concentrations of RORγ antagonists GSK805 and XY101 for 4 days. *n* = 3 biological replicates. **I** Immunoblotting analysis of the indicated proteins in RBE and HUCCT-1 cells treated with DMSO or the indicated RORγ antagonists. *n* = 3 biological replicates. All data from in vitro experiments shown above are the mean ± SD. *p < 0.05, **p < 0.01, ***p < 0.001.

**Fig. 3:**
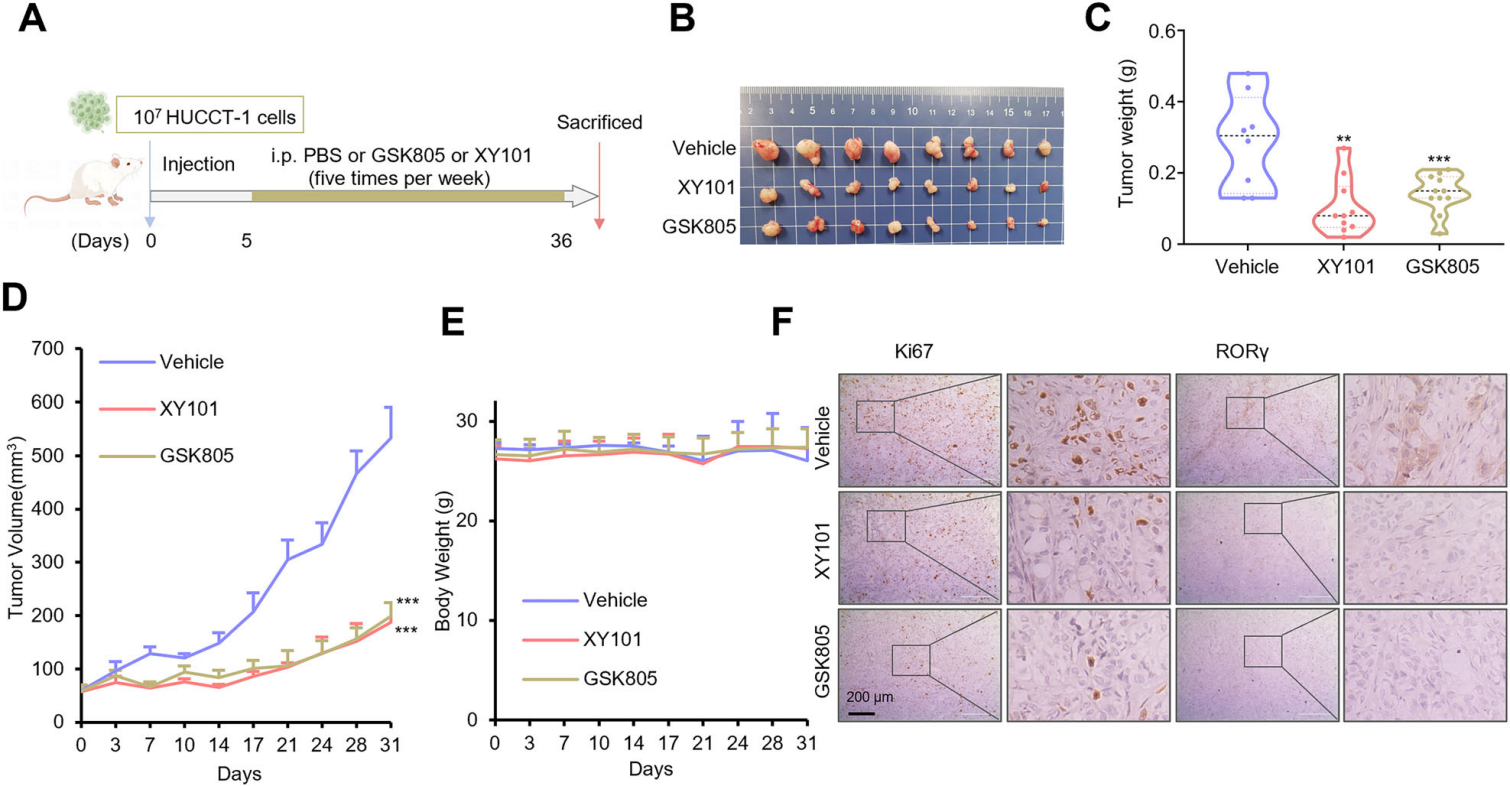
RORγ antagonists attenuate iCCA tumor progression. **A** Schematic depiction of HUCCT-1 subcutaneous xenografts establishment and treatment. **B**–**D** Male nude mice bearing HUCCT-1 subcutaneous xenografts received RORγ antagonists GSK805 (i.p., 5 mg/kg, *n* = 8), XY101 (i.p., 5 mg/kg, *n* = 8) or PBS (i.p., *n* = 8) five times per week for 31 days. Tumor volume was measured twice a week and mean tumor volume ± SEM is shown. Mean tumor weight ± SEM and representative photos of the tumors are shown. **E** Male nude mice bearing HUCCT-1 subcutaneous xenografts were administered RORγ antagonists GSK805 (i.p., 5 mg/kg, *n* = 8), XY101 (i.p., 5 mg/kg, *n* = 8), or PBS (*n* = 8) five times weekly for 31 days. During this period, body weights were measured twice weekly, and the mean body weight ± SEM is shown. **F** Immunohistochemistry images showing Ki67 and RORγ staining in randomly selected tumor tissues. Scale bar, 200 μm. All data shown from in vivo experiments shown above are the mean ± SEM. *p < 0.05, **p < 0.01, ***p < 0.001.

To further elucidate the role of RORγ in iCCA, we performed a series of functional studies. Knockdown of RORγ using two independent siRNAs significantly inhibited iCCA cells survival (Fig. 2A, B). RORγ knockdown also induced apoptosis, as evidenced by the activation of caspase-3 and caspase-7 along with an increased level of apoptosis-related protein markers, such as cleaved PARP-1 and cleaved caspase-7 (Fig. 2C, D). We next investigated the effects of pharmacological inhibition of RORγ using RORγ antagonists, GSK805 and XY101. Both compounds effectively inhibited cell growth at low micromolar concentrations, as assessed by cell viability and colony formation assays (Fig. 2E, F). Similar to the effects observed with *RORC* siRNA knockdown, pharmacological inhibition of RORγ suppressed cell proliferation, reduced colony formation, and promoted apoptosis in iCCA cells (Fig. 2G–I). To assess the impact of RORγ antagonists on tumor growth in vivo, we generated xenograft tumors in nude mice by implanting HUCCT-1 cells. Tumor-bearing mice were treated intraperitoneally with either vehicle or RORγ antagonists (5 mg/kg, five times a week; *n* = 8). The results showed that RORγ antagonists effectively suppressed tumor growth compared to the vehicle group. Importantly, antagonist treatment did not cause weight loss or histopathological changes in major organs (Fig. 3A–E and Supplementary Fig. S1A). IHC analysis of tumor sections revealed that RORγ antagonist treatment decreased Ki-67 staining and RORγ expression (Fig. 3F). Together, these results indicate that RORγ plays a crucial role in the growth and survival of iCCA, both in vivo and in vitro.

### RORγ directly regulates the transcription of FGF1 and FGFR2 in iCCA cells

**Fig. 4:**
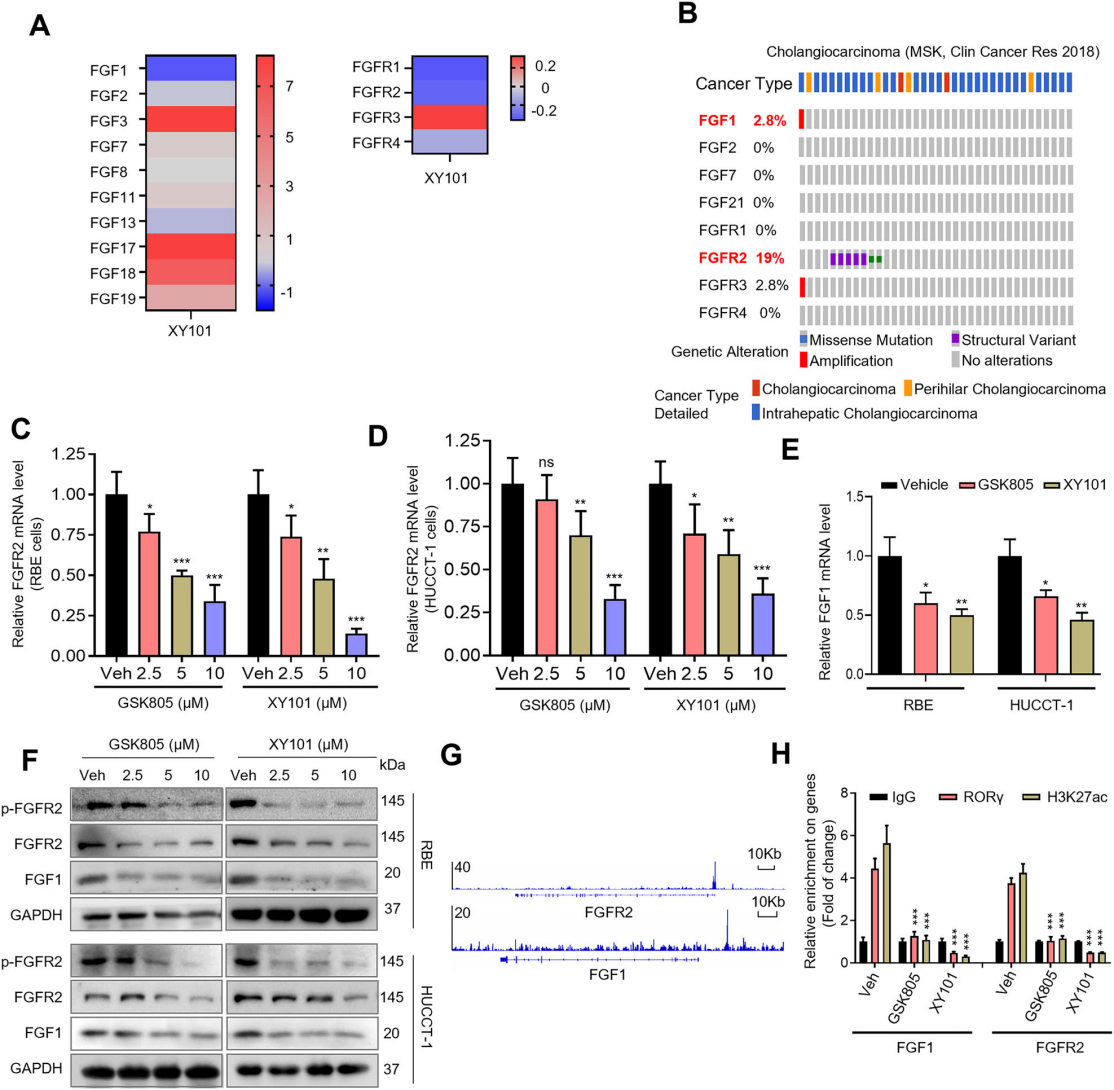
RORγ directly regulates the transcription of FGF1 and FGFR2 in iCCA cells. **A** Heatmap of mRNA expression changes of FGF family genes and FGFR family genes in HUCCT-1 cells treated for 2 days with 5 μM XY101. **B** Oncoprint display from cBioPortal of FGF family genes and FGFR family genes alterations in iCCA tumors. **C**, **D** qRT-PCR analysis of FGFR2 mRNA in iCCA cells treated with DMSO or RORγ antagonists (GSK805 and XY101) at indicated concentrations for 2 days. *n* = 3 biological replicates. **E** qRT-PCR assay of FGF1 mRNA in RBE and HUCCT-1 cells after treatment with DMSO, 5 μM GSK805 or XY101 for 2 days. *n* = 3 biological replicates. **F** Immunoblotting assay of indicated proteins in RBE and HUCCT-1 cells after treatment with DMSO, or RORγ antagonists (GSK805 and XY101) at indicated concentrations for 2 days. *n* = 3 biological replicates. **G** The genome browser illustrates RORγ-binding events on the promoters of the FGF1 and FGFR2 genes in triple-negative breast cancer cells, as previously reported. These findings are derived from our previous ChIP-seq dataset (GEO: GSE126380). **H** ChIP-qPCR analysis was performed to assess the relative enrichment of RORγ or H3K27ac at the promoters of the FGF1 and FGFR2 genes in iCCA cells treated with 5 μM GSK805 or XY101 for 2 days. The fold change indicates the enrichment of these factors at the gene promoters in response to GSK805 and XY101, normalized to the IgG enrichment in vehicle-treated cells, which was set as 1. All data from in vitro experiments shown above are the mean ± SD. *p < 0.05, **p < 0.01, ***p < 0.001.

To elucidate the mechanisms underlying how RORγ inhibition impairs cell survival and growth, we performed RNA sequencing (RNA-seq) on HUCCT-1 cells treated with 5 μM RORγ antagonist XY101 for two days, Transcriptomic profiling revealed 1063 genes that were significantly upregulated and 823 genes that were downregulated in response to XY101 treatment. Given the well-established role of the fibroblast growth factors (FGFs) and fibroblast growth factor receptors (FGFRs) signaling pathway in cancer progression, our findings show that FGF1 and FGFR2 were more significantly downregulated by antagonists compared with other ligands and receptors within the FGF/FGFR family (Fig. 4A). Public database analysis further confirmed that FGF1 and FGFR2 were frequently altered in iCCA, prompting us to focus on FGF1 and FGFR2 for subsequent mechanistic studies (Fig. 4B).

Considering the pivotal role of FGF1 and FGFR2 in iCCA oncogenesis and progression, we hypothesized that RORγ antagonists may attenuate iCCA malignancy via FGF/FGFR signaling. qRT-PCR analysis in RBE and HUCCT-1 cells demonstrated that treatment with RORγ antagonists suppressed the expression of FGF1 and FGFR2 (Fig. 4C–E). Immunoblotting further demonstrated that both antagonists significantly decreased the expression of FGF1, FGFR2 and phosphorylated FGFR2 (p-FGFR2) (Fig. 4F). Given the impact of RORγ inhibition on FGF1 and FGFR2, we sought to determine whether RORγ directly regulates their transcription. Analysis of our chromatin immunoprecipitation sequencing (ChIP-seq) data (GEO: GSE126380) revealed that RORγ specifically binds to the promoter regions of FGF1 and FGFR2 (Fig. 4G). ChIP-qPCR further confirmed RORγ binding at these loci and showed that antagonists treatment reduced both RORγ occupancy and the levels of active histone mark H3K27ac at these promoters in RBE and HUCCT-1 cells (Fig. 4H). These findings collectively demonstrate that RORγ acts as a direct transcriptional activator of both FGF1 and FGFR2 genes.

### FGF1 mediates RORγ-driven iCCA cells survival through FGFR2 activation

**Fig. 5:**
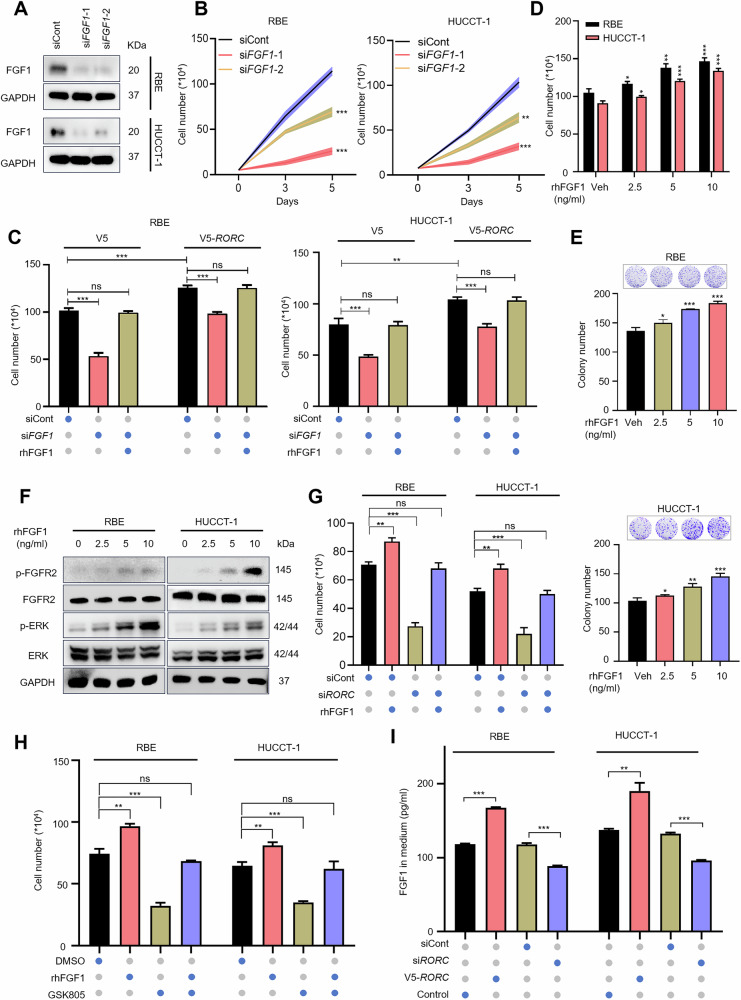
FGF1 mediates RORγ-driven iCCA cells survival through FGFR2 activation. **A** Immunoblotting analysis of FGF1 protein in iCCA cells transfected with *FGF1* siRNAs or control. *n* = 3 biological replicates. **B** RBE and HUCCT-1 cells were transfected with *FGF1* siRNAs or control. Live cells were counted at the indicated time points after transfection. *n* = 3 biological replicates. **C** Transfection of *FGF1* siRNAs or control into wild-type or RORγ-overexpressing RBE and HUCCT-1 cells, with or without 5 ng/ml human recombinant FGF1. Viable cells were counted after 4 days. *n* = 3 biological replicates. **D** RBE and HUCCT-1 cells were incubated with recombinant human FGF1 at the indicated concentrations for 4 days, after which live cells were counted. *n* = 3 biological replicates. **E** Colony formation assays were conducted to detect the survival of RBE and HUCCT-1 cells treated with recombinant human FGF1 at the indicated concentrations for 14 days. *n* = 3 biological replicates. **F** FGFR2, phosphorylated FGFR2 (p-FGFR2), and its downstream proteins were detected by immunoblotting in RBE and HUCCT-1 cells treated with recombinant human FGF1 at the specified concentrations for 20 min. *n* = 3 biological replicates. **G** Recombinant human FGF1 was added to iCAA cells transfected with *RORC* siRNAs or control, and live cells were counted after a 4-day incubation. *n* = 3 biological replicates. **H** Live cells were counted to detect the survival of RBE and HUCCT-1 cells treated with GSK805, either alone or in combination with human recombinant FGF1 at a concentration of 5 ng/ml. *n* = 3 biological replicates. **I** ELISA assay was performed to detect FGF1 levels in the supernatant of RORγ-overexpression and *RORC*-silenced iCAA cells. *n* = 3 biological replicates. All data presented above are shown as mean ± SD. *p < 0.05, **p < 0.01, ***p < 0.001.

FGF1 is known to play a key role in metabolic diseases and has potential therapeutic applications for conditions such as type 2 diabetes. Furthermore, FGF1 is implicated in cancer initiation and progression. Given our findings that RORγ promotes cell survival and upregulates FGF1 expression in tumor cells, we investigated whether FGF1 mediates RORγ-dependent survival in iCCA cells. Our results showed that silencing FGF1 markedly suppressed survival of RBE and HUCCT-1 cells (Fig. 5A, B). Furthermore, introducing exogenous recombinant human FGF1 (rhFGF1) rescued the inhibitory effects of FGF1 silencing (Fig. 5C). rhFGF1 treatment (ranging from 0 to 10 ng/mL) stimulated iCCA cells survival and colony formation in a dose-dependent manner (Fig. 5D, E). Given that FGF1 typically signals through FGFRs, and that FGF1 and FGFR2 expression are positively correlated in iCCA (Fig. S1B), we hypothesized that FGF1 acts through FGFR2.In both RBE and HUCCT-1 cells, rhFGF1 treatment induced FGFR2 phosphorylation and downstream ERK activation in a dose-dependent manner (Fig. 5F).In these cell lines, exogenous rhFGF1 treatment reversed the proliferation inhibition induced by RORγ knockdown with either *RORC* siRNA or RORγ antagonists, regardless of incubation conditions (Fig. 5G, H). ELISA quantification revealed that RORγ-overexpressing iCCA cells secreted higher levels of FGF1, whereas *RORC* knockdown reduced FGF1 secretion (Fig. 5I). Moreover, conditioned medium from RORγ-overexpressing HUCCT-1 cells significantly enhanced iCCA cells survival and colony formation (Supplementary Fig. S1C–E). Collectively, these findings demonstrate that RORγ promotes FGF1 secretion, which in turn enhances iCCA cells survival by activating FGFR2 signaling.

### Targeting RORγ overcomes resistance to pemigatinib in iCCA

**Fig. 6:**
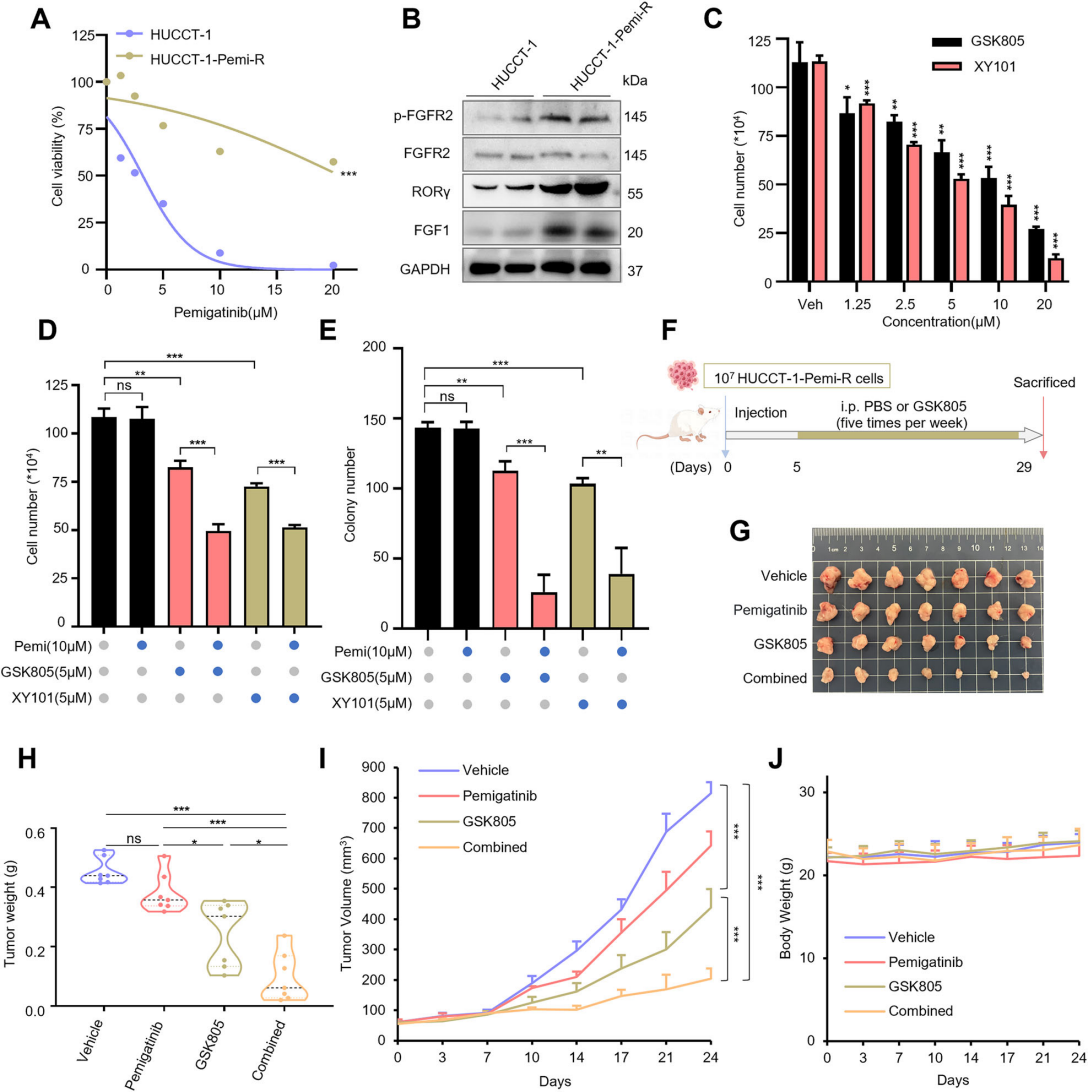
Targeting RORγ overcomes resistance to pemigatinib in iCCA. **A** Viability was performed to detect the sensitivity of HUCCT-1 and pemigatinib-resistant HUCCT-1(HUCCT-1-Pemi-R) cells treated with different concentrations of pemigatinib for 4 days. **B** Western blotting assay of the indicated proteins in HUCCT-1 and HUCCT-1-Pemi-R cells. **C** HUCCT-1-Pemi-R cells were treated with the indicated concentrations of RORγ antagonists GSK805 and XY101 or DMSO, live cells were counted. *n* = 3 biological replicates. **D**, **E** HUCCT-1-Pemi-R cells were treated with RORγ antagonists GSK805, XY101, and pemigatinib, either alone or in combination with GSK805 or XY101 and pemigatinib. Live cells counting and colony formation assay were conducted to evaluate cells survival. *n* = 3 biological replicates. **F** Schematic depiction of HUCCT-1-Pemi-R cells subcutaneous xenografts establishment and treatment. **G**, **H** Mice bearing HUCCT-1-Pemi-R xenografts were treated with PBS (*n* = 7), GSK805 (i.p., 5 mg/kg, *n* = 7), pemigatinib (i.p., 5 mg/kg, *n* = 7), or a combination of GSK805 and pemigatinib. Representative photos of the tumors were taken, and mean tumor weight ± SEM was shown. **I** Nude mice bearing HUCCT-1-Pemi-R subcutaneous xenografts were administered either a PBS (i.p., *n* = 7), GSK805 (i.p., 5 mg/kg, *n* = 7), pemigatinib (i.p., 5 mg/kg, *n* = 7), or a combination of GSK805 and pemigatinib five times a week. Tumor volume was measured twice a week and mean tumor volume ± SEM was shown. **J** Mice body weight was measured twice a week from different treatment groups described in (**I**) during the treatment period (presented as the mean ± SEM, *n* = 7). All data from in vitro experiments shown above are the mean ± SD and data shown from in vivo experiments are the mean ± SEM.*p < 0.05, **p < 0.01, ***p < 0.001.

Pemigatinib, a multi-FGFR inhibitor targeting FGFR1-3, is approved for iCCA treatment. Despite initial clinical responses, resistance develops in approximately 60% of patients within 6–9 months of continuous therapy. Emerging studies have indicated that RORγ is associated with therapeutic resistance in cancers, and pharmacological inhibition has shown promise for overcoming resistance in refractory malignancies [23–25]. This prompted us to investigate whether RORγ blockade could reverse pemigatinib resistance in iCCA. We tested the therapeutic potential of combining RORγ antagonists with pemigatinib in vitro and in vivo. First, we generated a pemigatinib-resistant cell line (HUCCT-1-Pemi-R) by progressively increasing pemigatinib concentrations over six months (Fig. 6A). Compared with the parental cells, these resistant cells exhibited elevated levels of RORγ, FGFR2, phosphorylated FGFR2, and FGF1 (Fig. 6B). Next, we examined whether blocking RORγ could resensitize the resistant cells to pemigatinib. Indeed, RORγ antagonists synergized with pemigatinib to inhibit the proliferation of HUCCT-1-Pemi-R cells (Fig. 6C–E). We then transplanted HUCCT-1-Pemi-R cells into nude mice and treated tumor-bearing mice with GSK805 and pemigatinib at 5 mg/kg (Fig. 6F). While GSK805 monotherapy suppressed tumor growth, pemigatinib alone exhibited no significant effect. Notably, cotreatment with GSK805 substantially potentiated the antitumor activity of pemigatinib (Fig. 6G–I). Importantly, neither monotherapy nor combination therapy affected body weight or major organs (Fig. 6J and Supplementary Fig. S1F). Together, the results suggest that combing RORγ antagonists with pemigatinib is a promising strategy to overcome resistance and improve iCCA treatment.

## Discussion

As the second most prevalent primary liver malignancy, iCCA is frequently diagnosed at advanced stages, precluding surgical intervention and leading to a poor prognosis. Current systemic therapies face dual challenges: immune checkpoint inhibitors show only limited efficacy, while chemotherapy is constrained by a suboptimal therapeutic index due to dose-limiting toxicities [40]. Recent advancements in targeted drug pemigatinib have improved outcomes for a subset of iCCA patients; however, its broader clinical utility is severely limited by the rapid emergence of resistance [10]. The slow pace of therapeutic innovation underscores the need to define resistance mechanisms and identify clinically actionable targets in iCCA. Here, we identify RORγ as a clinical-grade therapeutic target for iCCA. RORγ is highly expressed in iCCA tumors, and its inhibition, whether through pharmacological inhibition or *RORC* siRNA, effectively suppresses growth and restores sensitivity to pemigatinib. These effects are mediated through modulation of the RORγ-FGF1-FGFR2 axis, as demonstrated in vitro and in vivo.

RORγ plays essential roles in diverse biological processes, including Th17 cell differentiation and cytokine production, and considerable efforts have been devoted to developing RORγ-targeted therapies for autoimmune and metabolic diseases [21]. Accumulating evidence indicates that RORγ inhibition has therapeutic potential in psoriasis, with several compounds (e.g., VTP-43742, BI 730357, JTE-451) advancing to clinical trials [41, 42]. These reagents also present potential opportunities for treating other diseases, particularly cancers. In this study, we investigated the role of RORγ to uncover its new therapeutic utility in cancer. Pharmacological antagonism or siRNA-mediated knockdown of RORγ significantly inhibited iCCA cells survival. Since therapeutic resistance is a major barrier to targeted therapy in iCCA and RORγ has been implicated in modulating drug sensitivity across cancers (e.g., castration-resistant prostate cancer and osteosarcoma), these observations prompted further investigation into RORγ’s potential role in pemigatinib-resistant iCCA [23, 24]. RORγ expression was markedly elevated in pemigatinib-resistant cell lines. Notably, combination therapy with RORγ antagonists and pemigatinib produced synergistic antitumor activity, inhibiting tumor progression in pemigatinib-resistant xenograft models. Collectively, these findings highlight RORγ as a promising therapeutic target in iCCA.

Our study demonstrated that treatment with RORγ antagonists (GSK805 and XY101) significantly downregulated the expression of FGF1 and FGFR2 within the FGF/FGFR signaling pathway, as confirmed by gene expression profiling. FGF1 functions as a key metabolic regulator that governs glucose homeostasis and insulin sensitivity, and has shown therapeutic potential for type 2 diabetes mellitus [43–45]. Accumulating evidence indicates that FGF1 also plays a critical role in mitogenic signaling and therapeutic resistance in various malignancies [46, 47]. Besides iCCA, FGF1 sustains the survival of dormant cancer cells in breast cancer, modulates senescence-associated malignancy in prostate cancer, and reactivates RAF-MEK signaling in pancreatic ductal adenocarcinoma to bypass targeted inhibition [48–50]. In our study, both FGF1 siRNA and RORγ antagonists reduced FGF1 expression and suppressed tumor cell survival. Conversely, exogenous FGF1 significantly enhanced FGFR2 signaling activation and promoted oncogenic effects.

Pemigatinib, a selective FGFR1-3 inhibitor, is approved by the FDA for the treatment of advanced cholangiocarcinoma with FGFR2 fusions or rearrangements. While efficacy in this biomarker-defined subgroup is well established, emerging clinical data suggest potentially therapeutic activities in broader populations. For example, the pivotal FIGHT-202 trial (NCT02924376) reported partial responses in a small subset of cholangiocarcinoma patients harboring non-FGFR2 alterations (e.g., other FGF/FGFR pathway abnormalities) or even no detectable FGF/FGFR alterations. However, these observations were not statistically significant, and the clinical benefit in these groups remains uncertain due to low response rates and short durations of disease control [39, 51].These suggest potential therapeutic activities beyond the current biomarker-defined populations [51]. The partial efficacy observed in patients without FGFR2 fusions or rearrangements may be attributed to drug resistance, as existing paradigms primarily emphasize alterations with the FGFR2 kinase or transmembrane domains. Our study identifies RORγ as a key regulator of pemigatinib resistance through dual mechanisms: it directly regulates FGFR2 transcription and induces FGF1 transcription and secretion, collectively sustaining FGFR2 signaling. Together, our findings establish the RORγ-FGF1-FGFR2 axis as both a driver of therapeutic resistance and a druggable vulnerability in iCCA. Key advances include: (i) the first demonstration that the RORγ-FGF1-FGFR2 axis mediates adaptive resistance to pemigatinib, and (ii) preclinical validation that simultaneously targeting RORγ and FGFR2 yields synergistic antitumor activity in pemigatinib-resistant models. These findings challenge the mutation-centric paradigm of resistance and facilitate the development of biomarker-independent therapeutic strategies. However, the non-genomic adaptive mechanisms mediated by RORγ remain poorly defined [10, 52], and this study focused on the tumor-intrinsic role of RORγ in iCCA cells, without exploring the potential role of the RORγt isoform within the tumor microenvironment.

## Conclusion

This study demonstrates that RORγ transcriptionally regulates FGF1 and FGFR2, thereby promoting FGF1 secretion and FGFR2-mediated downstream signaling. Furthermore, it elucidates the critical role of the RORγ-FGF1-FGFR2 axis in iCCA progression and pemigatinib resistance. Given that RORγ is a clinically validated target with inhibitory agents in active clinical trials, its inhibition represents a promising therapeutic strategy for iCCA.

## Materials and methods

### Cell culture

RBE and HUCCT-1 cells were cultured in RPMI-1640 medium. All cells were authenticated by STR profiling within the past 6 months and confirmed to be mycoplasma free. To generate the pemigatinib-resistant HUCCT-1-Pemi-R cells, HUCCT-1 cells were initially cultured in 1 µM pemigatinib. After achieving stable growth, the pemigatinib concentration was gradually increased during cell passage until a stable, resistant population emerged in the presence of a high drug concentration, a process that required approximately six months. All culture media were supplemented with 10% fetal bovine serum (Exocell) and 1% penicillin/streptomycin (Gibco). All cells were maintained at 37 °C in a incubator with 5% CO_2_.

### Chemicals

The sources of chemicals are as follows: GSK805, and XY101 were obtained from WuXi AppTec (China); Pemigatinib was purchased from TargetMol (USA); Recombinant human FGF1 was obtained from MedChemExpress (USA).

### siRNA transfection

Cells were seeded into 6-well plates at 1 ×10^5^ cells per well and cultured overnight to reach a confluence of 60–70%, and then transfected with synthetic siRNAs using DharmaFECT (Dharmacon, USA) in Opti-MEM (Invitrogen, USA) at a final concentration of 20 nM. Cells were harvested at the indicated time points for subsequent assays. The siRNA sequences are shown in Supplementary Table 1.

### Cell viability

Cells were seeded in 96-well plates at 1500 cells per well in 100 µL of culture media. Twenty-four hours later, 50 µL of serially diluted compounds (prepared in culture medium) was added to each well. After an additional 4 days of incubation, Cell-Titer GLO reagents (Promega Corporation, Madison, USA) were added, and luminescence was measured on a GLOMAX microplate luminometer (Promega Corporation, Madison, USA) in accordance with the manufacturer’s instructions. Cell viabilities were normalized to vehicle-treated controls, which were set at 100%.

### Patient specimens

Primary iCCA tumors and non-tumor gallbladder tissue specimens, fixed in paraffin, were collected from The Third Affiliated Hospital of Southern Medical University and The First People’s Hospital of Foshan. All cases were pathologically confirmed, with approval from the hospital’s institutional review board (N202504-08).

### Immunohistochemistry (IHC)

Tissue sections were incubated at 60 °C for one hour, deparaffinized and rehydrated through graded ethanol. Antigen retrieval was conducted in pH6.0 citric acid buffer (Servicebio, China) using microwave heating. Peroxidase activity was blocked with hydrogen peroxide, and non-specific binding was blocked with goat serum. The sections were subsequently incubated overnight with a primary antibody at 4 °C, followed by a one-hour incubation with a secondary antibody at room temperature the following day. Protein expression was visualized using a DAB kit (Beyotime, China), and sections were counterstained with hematoxylin and mounted. Finally, images were acquired using the EVOS FL Auto (USA). All antibodies used in this study are described in Supplementary Table 2.

### Caspase-3/7 activity and cell growth

Caspase-3/7 activity was measured using the caspase-Glo 3/7 assay kit (Promega Corporation, USA) according to the manufacturer’s instructions, and results were standardized to the protein concentration. For cell growth assays, cells were seeded in 6-well plates at 1 × 10^5^ cells per well and treated as indicated. Total viable cells were counted with a cell counter.

### Colony formation

RBE, HUCCT-1 or HUCCT-1-Pemi-R cells were seeded in 6-well plates and cultured for 14 days, with the medium changed and compounds treatment every 3 days. Then, colonies were fixed with 4% paraformaldehyde (Beyotime, China) for 15 min, washed three times with PBS, and stained with crystal violet (Beyotime, China) for 15 min. The number of colonies was quantified after washing three times with PBS.

### Quantitative reverse transcription polymerase chain reaction (qRT‒PCR)

Total RNA was extracted using TRIzol (Yeasen Biotechnology, China), and cDNA was prepared according to the manufacturer’s protocol. qRT‒PCR was performed using SYBR Green Supermix (Dongsheng Biotech, China) on a CFX96^TM^ Real-Time System (Bio-Rad, CA, USA). The experiments were performed at least three times independently. Primers sequences for the qRT‒PCR are shown in Supplementary Table 3.

### Chromatin immunoprecipitation (ChIP)-qPCR

Cells were fixed with paraformaldehyde in the culture medium, and crosslinking was quenched with glycine. Cells were harvested by centrifugation, resuspended, and lysed in Farnham lysis buffer. After centrifugation, the pellet was resuspended in RIPA buffer and subjected to ultrasonication for 15 min using the Covaris E220 according to the manufacturer’s instructions. Subsequently, a small aliquot of the cell lysate was mixed overnight with Dynabeads™ Protein G magnetic beads pre-incubated with the target antibody. DNA fragments were purified using the GeneJET PCR Purification Kit (Thermo Scientific, K0702, USA) and subsequently analyzed by real-time PCR. The primer sequences for ChIP-qPCR are listed in Supplementary Table 4.

### Western blotting analysis

Cells were lysed in RIPA lysis buffer supplemented with protease inhibitors. Protein concentrations were quantified using a BCA assay kit (Beyotime, China). Equal amounts of protein were separated by Tris-glycine SDS‒PAGE and transferred to PVDF membranes (EMD Millipore, ISEQ00010). Membranes were blocked with 5% skim milk, incubated overnight at 4 °C with primary antibodies, and then with HRP-conjugated secondary antibodies (Cell Signaling Technology, USA). Western blotting analysis was performed using antibodies specifically against RORγ and other indicated proteins. All antibodies used in this study are described in Supplementary Table 2.

### RNA sequencing analysis

HUCCT-1 cells were treated with vehicle, or XY101 (5 µM) for 2 days. Total RNA was extracted, and RNA sequencing (RNA-seq) libraries were constructed using the Illumina TruSeq Kit. RNA-seq was performed by Beijing Genomics Institute (BGI, Shenzhen, China). Briefly, poly(A)⁺ mRNA was enriched using oligo(dT) magnetic beads. cDNA was synthesized under high-temperature conditions and purified according to the kit protocol. The reads were aligned to the reference human genome assembly (GRCh37/hg19) using BWA and Bowtie 2. Gene expression levels were quantified from the RNA-seq data using RSEM, and a heatmap was subsequently generated to visualize the expression profiles.

### Enzyme linked immunosorbent assay (ELISA)

The concentration of FGF1 in culture supernatants was measured using an ELISA kit (ELK Biotechnology, China). First, the cell supernatant was centrifuged at 1000 × *g* for 20 min to remove impurities and cellular debris. The clarified supernatant was then transferred to a pre-coated microplate (100 μL per well) and incubated at 37 °C for 80 min. After washing, 100 μL of biotinylated antibody working solution was added and incubated at 37 °C for 50 min. Following another wash, 100 μL of horseradish peroxidase (HRP) enzyme working solution was introduced, and the incubation was continued for 50 min at 37 °C. After the final wash, 90 μL of TMB substrate was added and incubated at 37 °C for 20 min. The reaction was stopped by adding 50 μL of stop solution, and absorbance was measured at 450 nm immediately.

### Bioinformatics analysis

The gene expression dataset GSE138709 was obtained from the Gene Expression Omnibus (GEO) database. Data processing was conducted using R (version 3.4; Bioconductor) with the edgeR package. Of note, the expression and distribution of *RORC* were analyzed specifically in tumor samples, whereas all other analyses utilized both tumor and adjacent normal tissues. Gene alterations were analyzed using cBioPortal for Cancer Genomics.

### Animal experiments

Four-week-old male NOD/SCID mice (average body weight ~18 g) were purchased from Nanjing Biomedical Research Institute of Nanjing University (Nanjing, China). To generate a xenograft model, exponentially growing HUCCT-1 or HUCCT-1-Pemi-R cells (1 × 10^7^ cells) were suspended in 100 μL of PBS and Matrigel (1:1) and injected subcutaneously into the flank of the mice. When the average estimated tumor volume reached ~50 mm^3^, mice were grouped randomly with comparable tumor volume. Tumor volume and body weight were measured twice weekly. Tumor volume was calculated using the equation V = π/6 (length × width^2^). At the end of the experiment, the mice were sacrificed. Tumors were harvested, weighed and processed for immunohistochemistry. All procedures were approved by the Institutional Animal Care and Use Committee of Sun Yat-sen University (SYSU-IACUC-2023-001164). All in vivo experiments employed group sizes of *n* ≥ 5 animals. Blinding was maintained during group allocation, data collection, and analysis.

### Statistical analysis

All statistical analyses were conducted with GraphPad Prism 9.5 (GraphPad Software, USA). As for in vitro experiment, results are presented as mean ± SD from three independent experiments. For in vivo studies, tumor volume and tumor weight are presented as mean ± SEM. Statistical significance was evaluated using a two-tailed Student’s *t* test for comparisons between two groups, and one-way analysis of variance (ANOVA) was used for comparisons between three or more groups. *p* values < 0.05 were considered to be significant. *p* values were calculated for *p < 0.05, **p < 0.01, ***p < 0.001.

## Supplementary information

Supplemental Material 1

Supplemental Material 2

## Data Availability

The raw RNA-seq data reported in this paper have been deposited in Gene Expression Omnibus with GEO accession GSE306923. The single-cell RNA-seq dataset (GSE138709) of intrahepatic cholangiocarcinoma (ICC) was obtained from the Gene Expression Omnibus (GEO) database (https://www.ncbi.nlm.nih.gov/geo/). All associated data supporting this research are available upon reasonable request from the lead contact.

## Code availability

This paper does not involve the creation of any original code.
