# Supplementary material for: FGF1-FGFR2 axis regulated by nuclear receptor RORγ represents an effective strategy in intrahepatic cholangiocarcinoma
Source: Cell Death Discov. 2025 Dec 22;11:562. doi: 10.1038/s41420-025-02844-8 (PMC12722201; doi:10.1038/s41420-025-02844-8)
Supplement: Supplementary file 1 — Supplemental Material 1 [file 41420_2025_2844_MOESM1_ESM.docx]

**FGF1-FGFR2 axis regulated by nuclear receptor RORγ represents an effective strategy in intrahepatic cholangiocarcinoma**

**Zhanfeng Gu^1,8^, Xiaojuan Wang^3,8^, Hong Wang^2,8^ Junhua Wang^4,8^, Zhaorong Huang^1^, Dongyue Pan^7,1^, Zhenhua Zhang^2^, Yechun Zeng^2^, Guodi Cai^2^, Huizi Sun^2^, Jun Zheng^1^, Yichu Nie^4^, Qingwen Zhang^5^, Haolong Li^6^，Franky Leung Chan^6^, Junjian Wang^2, *^, Jianwei Zheng ^2, *^, Yingfang Fan^1,*^**

^1^ Department of Hepatobiliary surgery, The Third Affiliated Hospital, Southern Medical University, Guangzhou 510630, Guangdong, China.

^2^ National-Local Joint Engineering Laboratory of Druggability and New Drugs Evaluation, School of Pharmaceutical Sciences, Sun Yat-sen University, Guangzhou 510006, Guangdong, China.

^3^ Hepato-Pancreato-Biliary Center, Beijing Tsinghua Changgung Hospital, Key Laboratory of Digital Intelligence Hepatology (Ministry of Education), School of Clinical Medicine, Tsinghua Medicine, Tsinghua University, Beijing 102218, China.

^4^ Clinical Research Institute, The First People’s Hospital of Foshan, Foshan 528000, Guangdong, China.

^5^ State Key Laboratory of Quality Research in Chinese Medicine and Institute of Chinese Medical Sciences, University of Macau, Taipa, Macao.

^6^ School of Biomedical Sciences, The Chinese University of Hong Kong, Sha Tin, Hong Kong.

^7^ The Breast Center, Cancer Hospital of Shantou University Medical College, Shantou 515041, Guangdong, China.

^8^ These authors contributed equally: Zhanfeng Gu, Xiaojuan Wang, Hong Wang, and Junhua Wang.

**^*^** Correspondence: wangjj87@mail.sysu.edu.cn (Junjian Wang), zhengjw23@alumni.sysu.edu.cn (Jianwei Zheng), fanxifan@smu.edu.cn (Yingfang Fan).

**
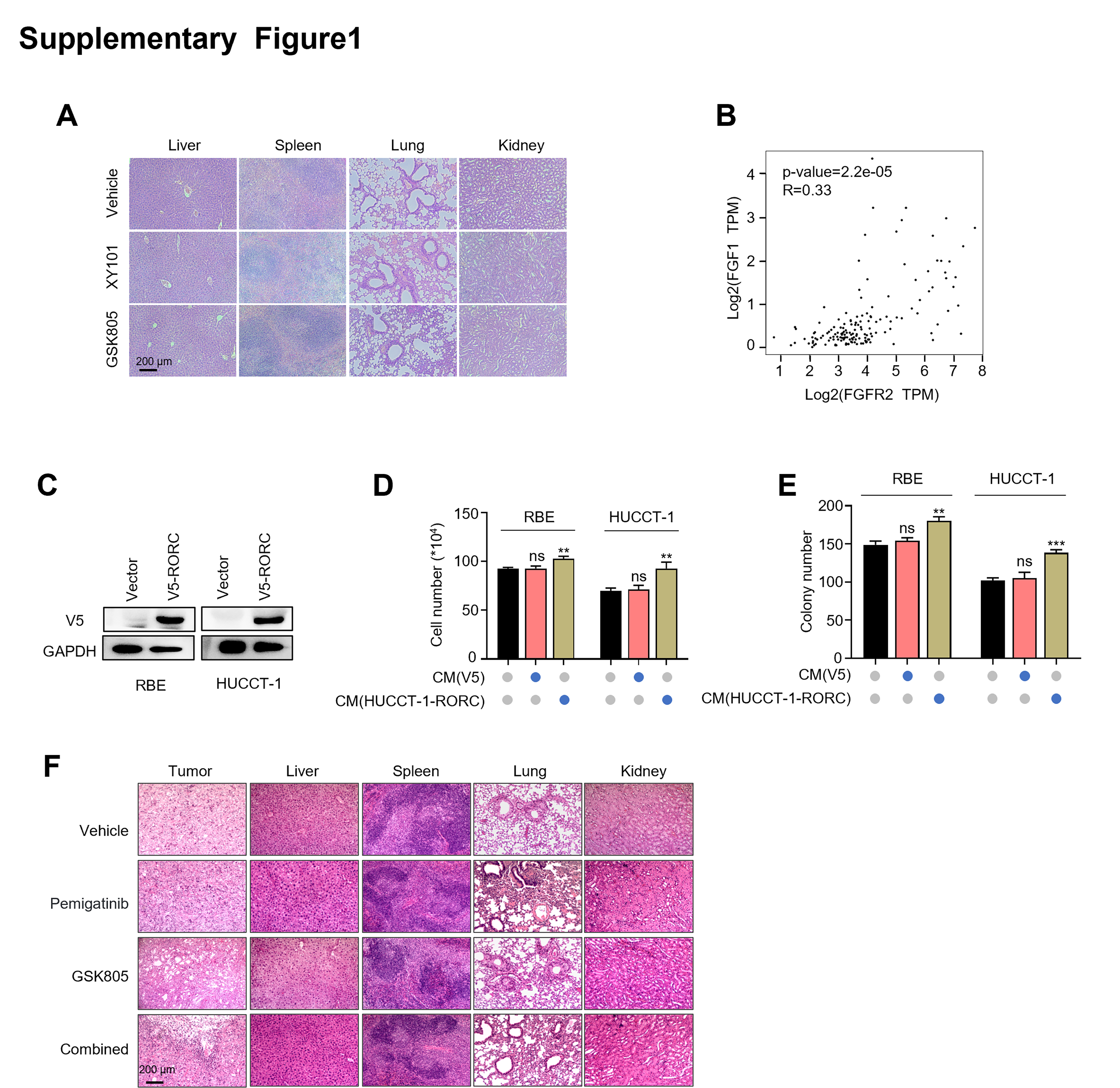
**

**Supplementary Figure 1. Functional validation of RORγ's role in iCCA tumorigenesis.**

**A** Representative H&E-stained tumor sections and major organs histology (liver, spleen, lung, kidney) from HUCCT-1 xenografts. Scale bar, 200 μm.

**B** Correlation of FGF1 and FGFR2 mRNA expression in iCCA.

**C** Western blotting of the indicated proteins after RORγ-overexpression in iCCA cells. *n*=3 biological replicates.

**D** HUCCT-1 and RBE cells receiving treatment with conditioned medium from wild type and RORγ-overexpression HUCCT-1 cells, and live cells were counted. *n*=3 biological replicates.

**E** Colony formation assays were performed in iCCA cells receiving treatment with conditioned medium from wild type and RORγ-overexpression HUCCT-1 cells. *n*=3 biological replicates.

**F** H&E staining of tumor sections and major organs from HUCCT-1-Pemi-R bearing mice is shown. Scale bar, 200 μm.

All data from *in vitro* experiments shown above are the mean ± SD and data shown from *in vivo* experiments are the mean ± SEM.**p* < 0.05, ***p* < 0.01, ****p* < 0.001.

**Supplementary Table 1. Primer sequences for siRNA transfection.**

| Oligo Name | Sequence（5'-3'） | |
| --- | --- | --- |
|  |  |  |
| si-RORC-1 | Forward | CGAGGAUGAGAUUGCCCUCUATT |
|  | Reverse | UAGAGGGCAAUCUCAUCCUCGTT |
| si-RORC-2 | Forward | GCCCUCAUAUUCCAACAACUUTT |
|  | Reverse | AAGUUGUUGGAAUAUGAGGGCTT |
| si-FGF1-1 | Forward | GCCCUGACCGAGAAGUUUAAUdTdT |
|  | Reverse | AUUAAACUUCUCGGUCAGGGCdTdT |
| si-FGF1-2 | Forward | GAGAAGUUUAAUCUGCCUCCAdTdT |
|  | Reverse | UGGAGGCAGAUUAAACUUCUCdTdT |
| siCont | Forward | CAGTCGCGTTTGCGACTGG |
|  | Reverse | CCAGTCGCAAACGCGACTG |

**Supplementary Table 2. Antibodies for wb and IHC.**

| Antibodies | Source | Identifier | Dilution |
| --- | --- | --- | --- |
| RORγ | Proteintech | 29910-1-AP | 1:200(IHC) |
| RORγ | Proteintech | 29910-1-AP | 1:500(wb) |
| GAPDH | Cell Signaling Technology | #2118 | 1:1000 |
| PARP | Cell Signaling Technology | #9542 | 1:1000 |
| cleaved-Caspase7 | Cell Signaling Technology | #9491 | 1:1000 |
| FGF1 | Cell Signaling Technology | #26526 | 1:1000 |
| p-FGFR2 | Cell Signaling Technology | #3471S | 1:1000 |
| FGFR2 | Cell Signaling Technology | #23328 | 1:1000 |
| ERK | Cell Signaling Technology | #4695 | 1:1000 |
| p-ERK | Cell Signaling Technology | #5683 | 1:1000 |
| Anti-mouse IgG | Cell Signaling Technology | #7076 | 1:5000 |
| Anti-rabbit IgG | Cell Signaling Technology | #7074 | 1:5000 |

**Supplementary Table 3. Primer sequences for qRT-PCR.**

| Genes | Sequence（5'-3'） | |
| --- | --- | --- |
|  |  |  |
| FGF1 | Forward | ATGGCACAGTGGATGGGACAAG |
|  | Reverse | TAAAAGCCCGTCGGTGTCCATG |
| FGFR2 | Forward | CGCTGGTGAGGATAACAACACG |
|  | Reverse | TGGAAGTTCATACTCGGAGACCC |
| β-Actin | Forward | GAGAAAATCTGGCACCACACC |
|  | Reverse | ATACCCCTCGTAGATGGGCAC |

**Supplementary Table 4. Primer sequences for ChIP-qPCR assay.**

| Genes | Sequence（5'-3'） | |
| --- | --- | --- |
|  |  |  |
| FGF1 | Forward | TAAAGGCAGGCTCAAACCCT |
|  | Reverse | GCAAAGAACACACTGCCTCC |
| FGFR2 | Forward | ATCGCTCTGGTGGAGAGAGG |
|  | Reverse | GGCAGTGGACAGCCAATAAC |
