# Supplementary material for: FGF1-FGFR2 axis regulated by nuclear receptor RORγ represents an effective strategy in intrahepatic cholangiocarcinoma
Source: Cell Death Discov. 2025 Dec 22;11:562. doi: 10.1038/s41420-025-02844-8 (PMC12722201; doi:10.1038/s41420-025-02844-8)

Figure 2A

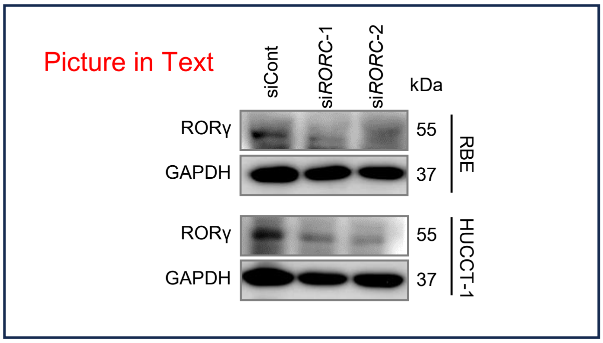

Original wb picture

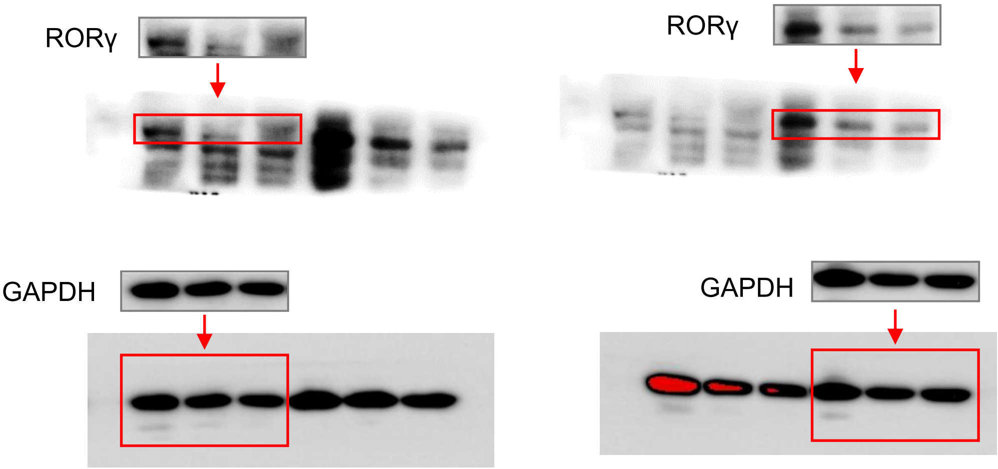

Figure 2D

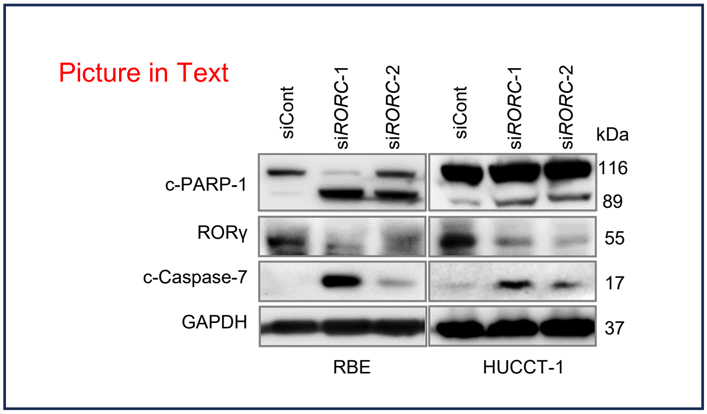

Original wb picture

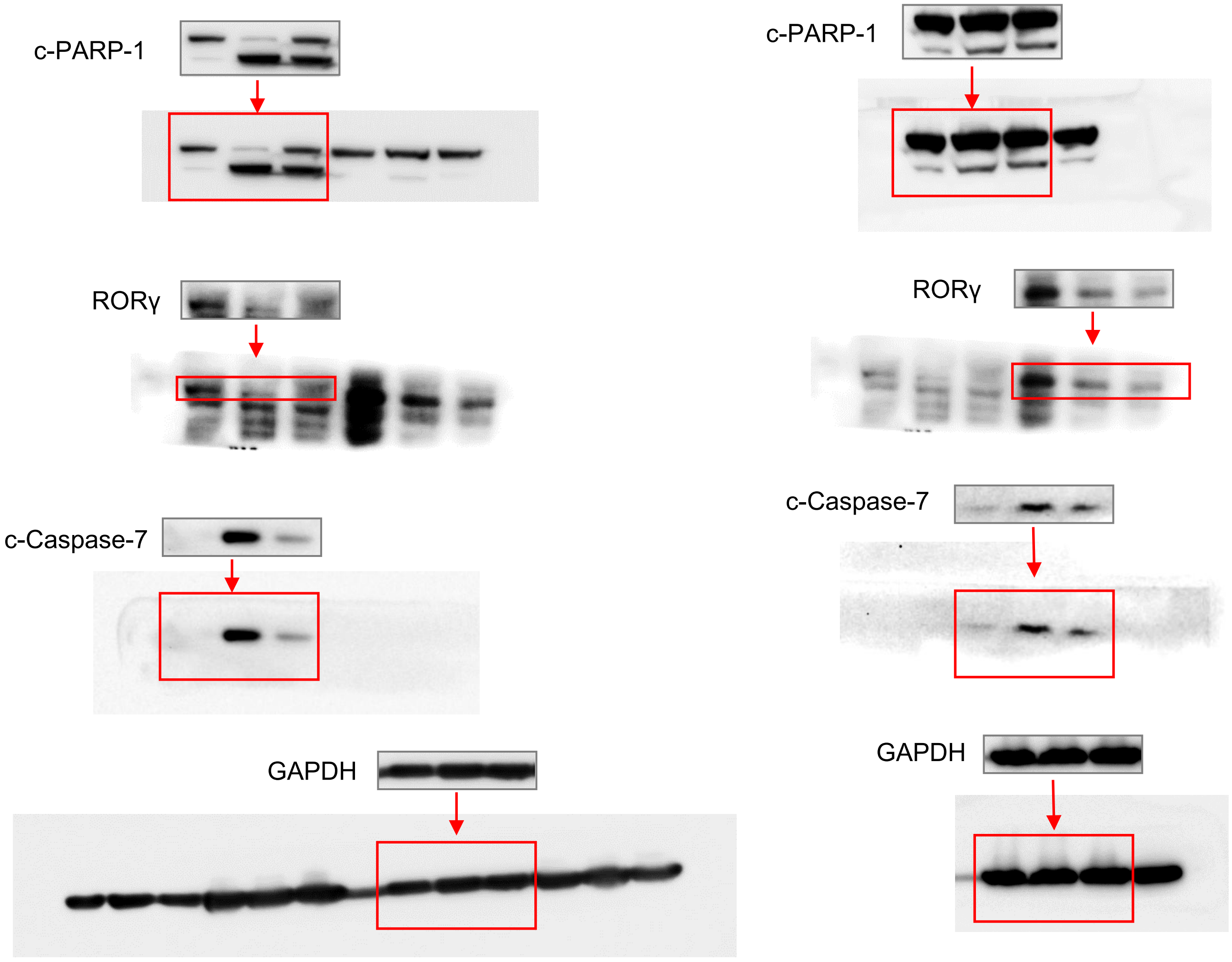

Figure 2I

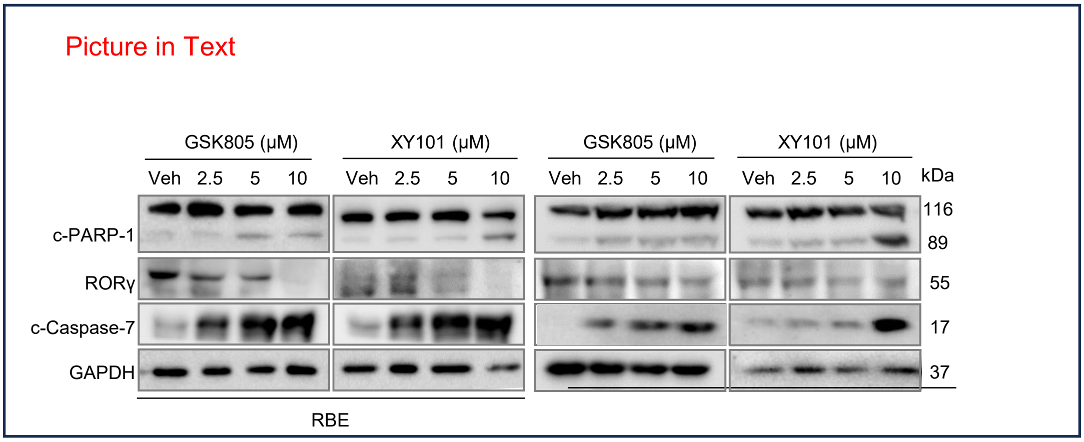

Original wb picture(RBE)

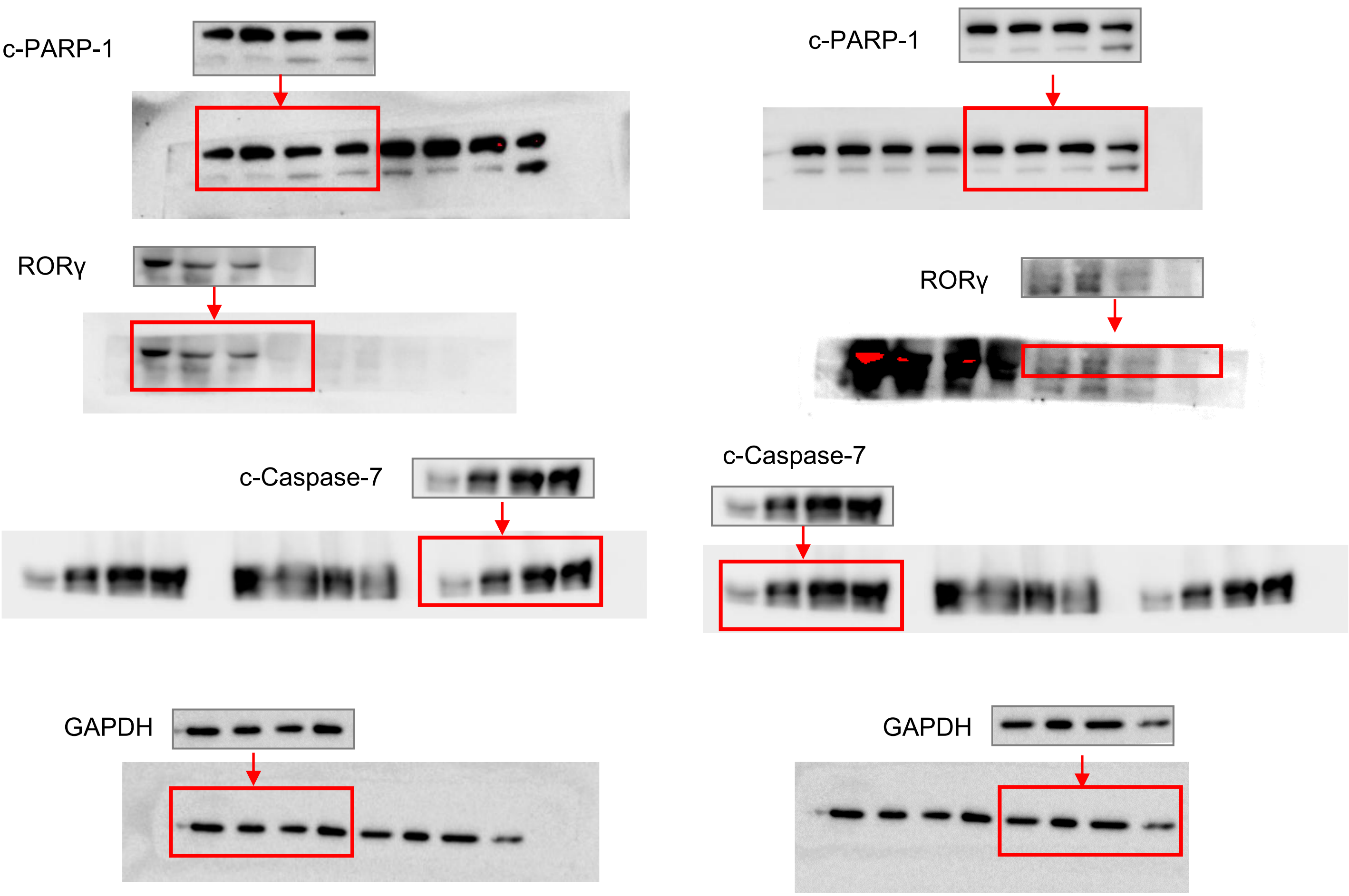

Figure 2I

Picture in Text

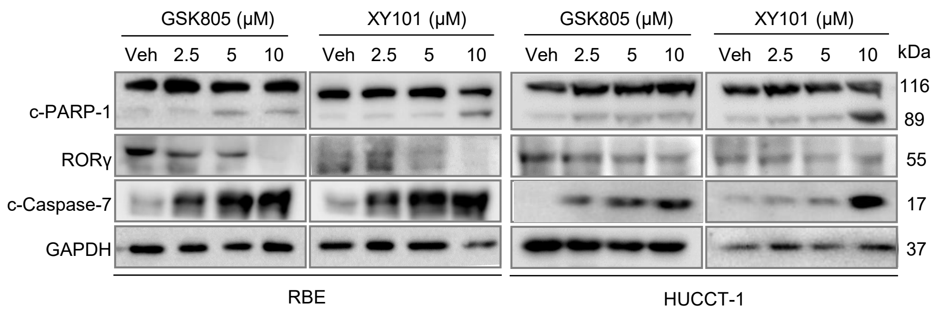

Original wb picture(HUCCT-1)

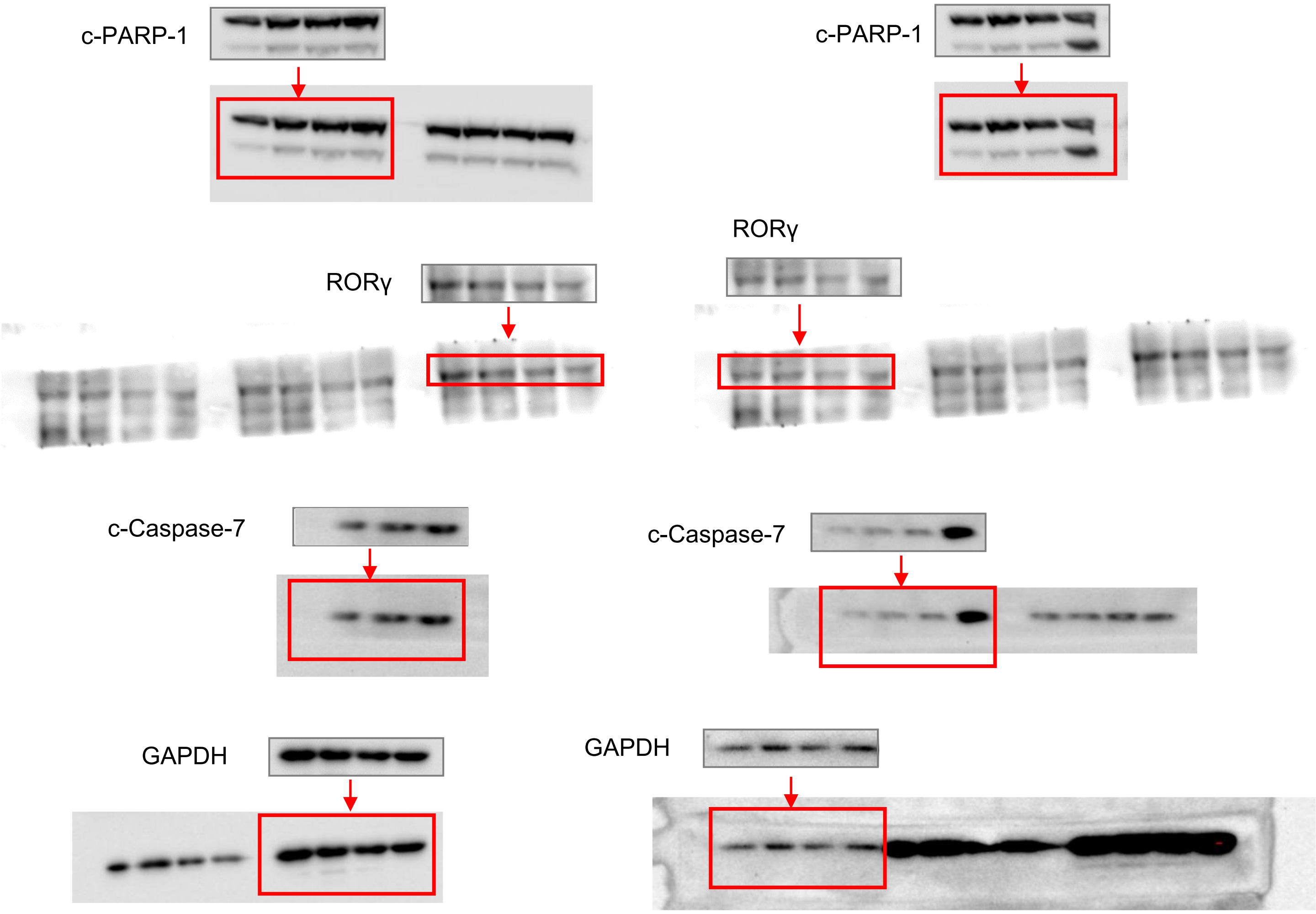

Figure 4G

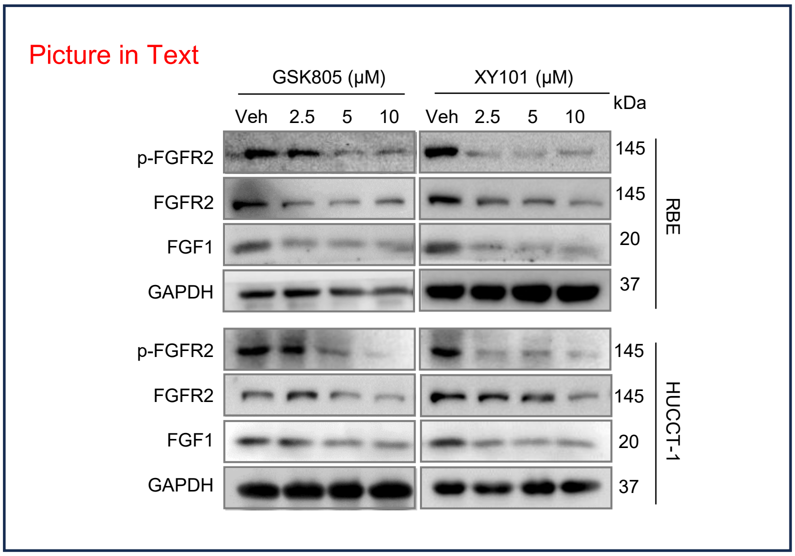

Original wb picture(RBE)

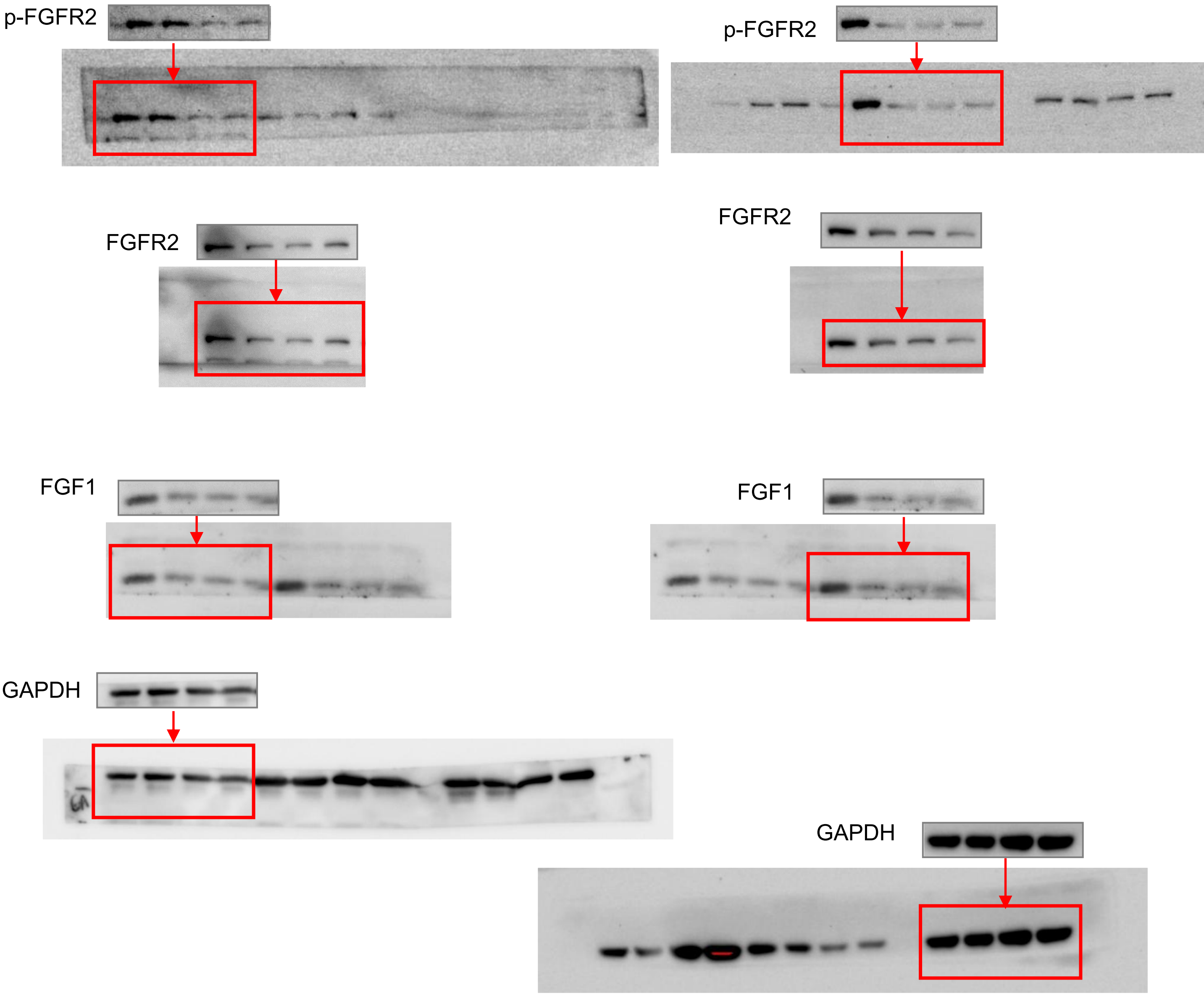

Figure 4G

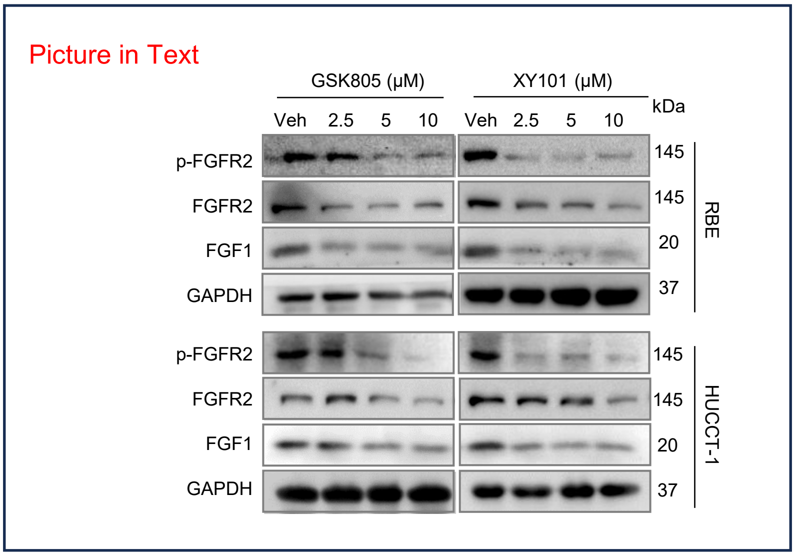

Original wb picture(HUCCT-1)

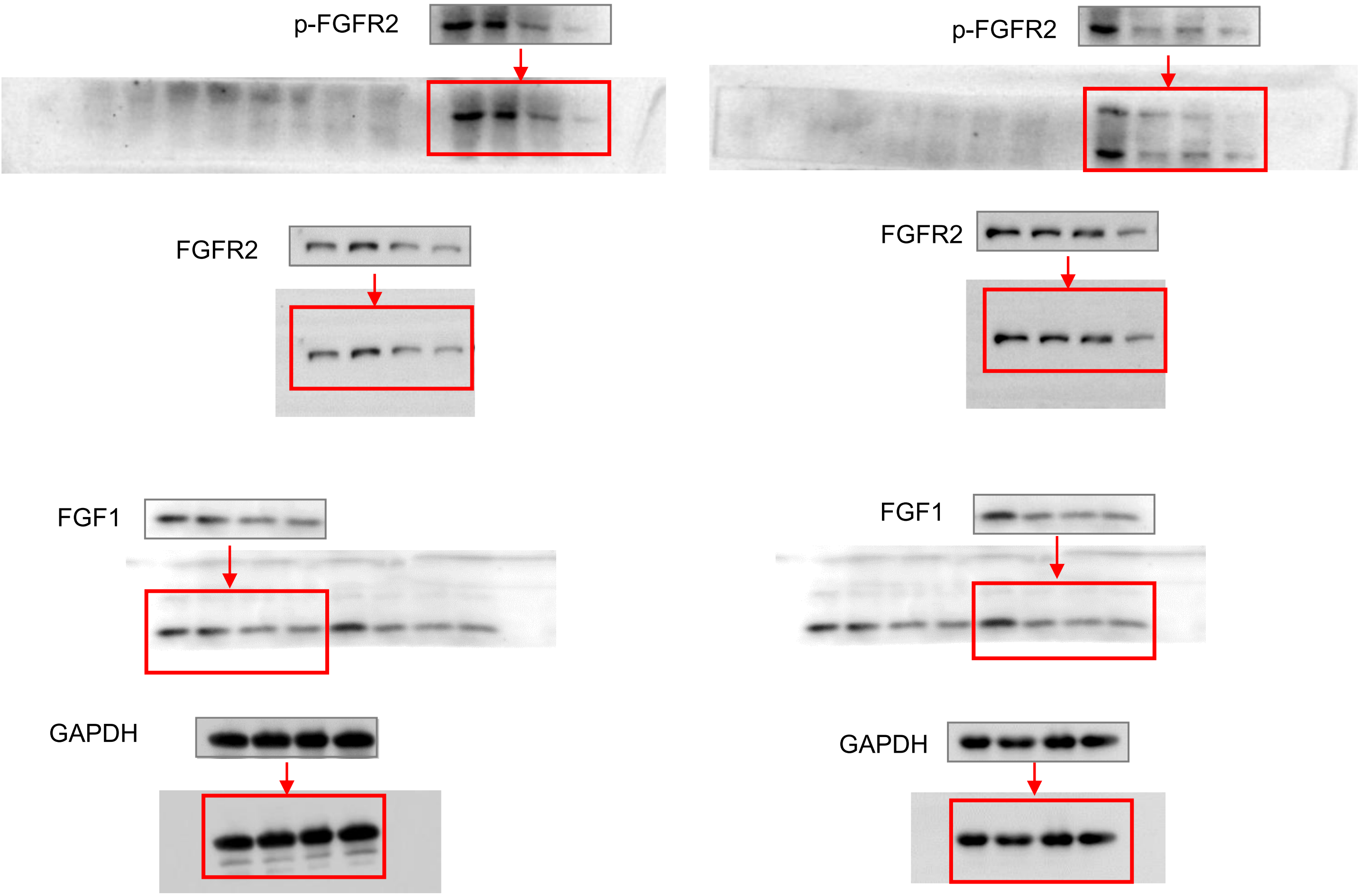

Figure 5A

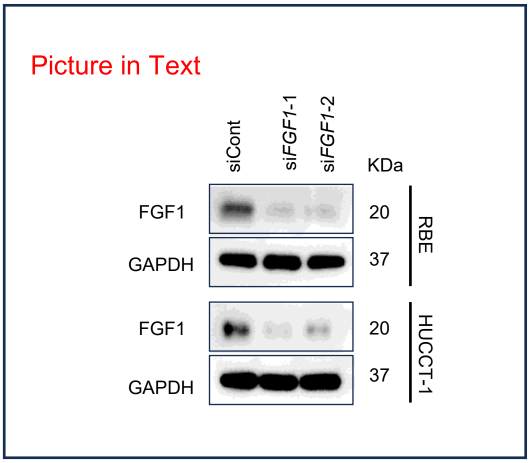

Original wb picture

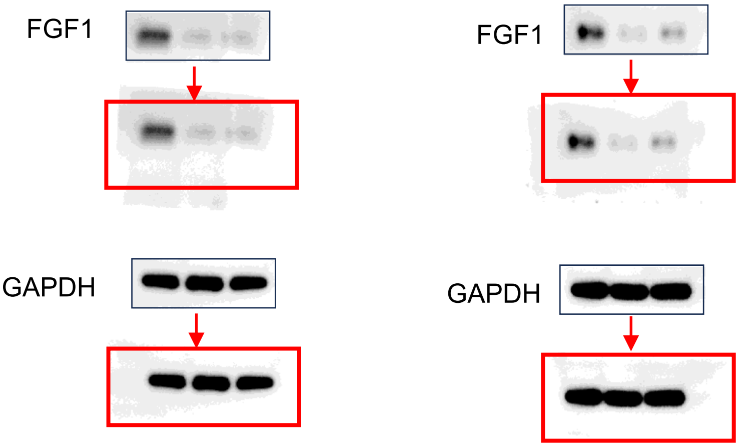

Figure 5F

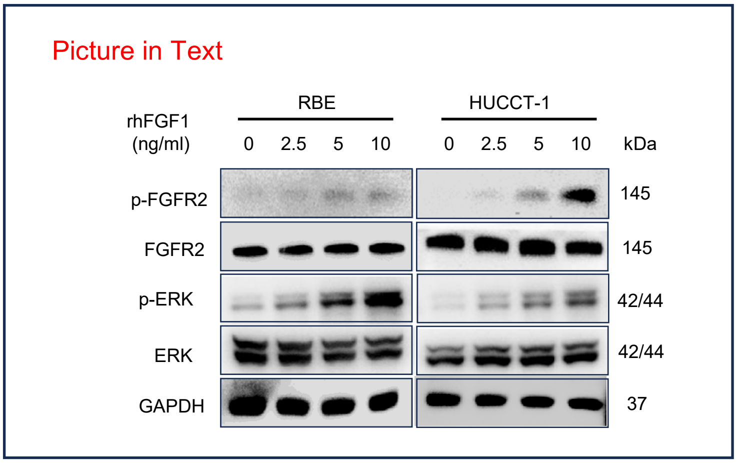

Original wb picture

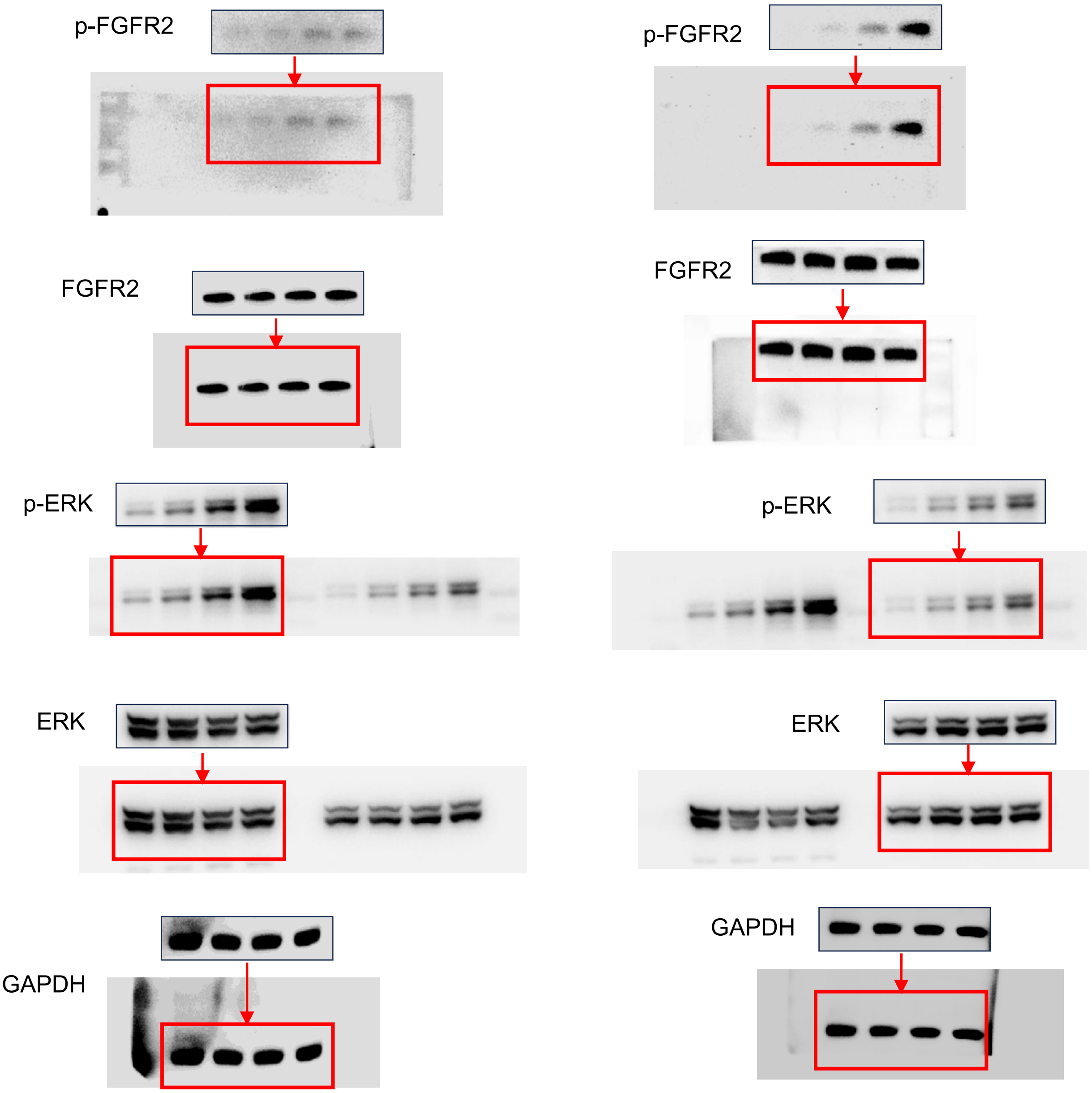

Figure 6B

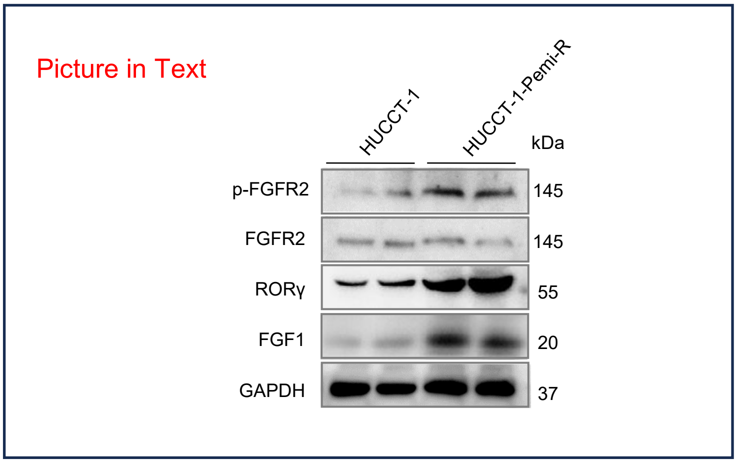

Original wb picture

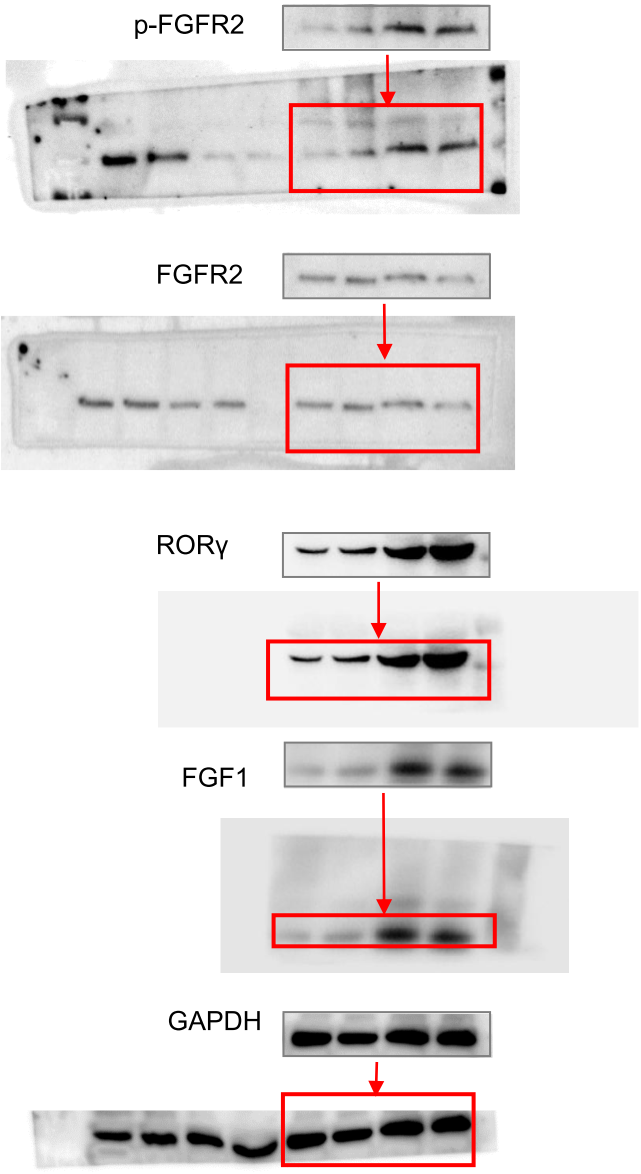

Supplementary Figure 1

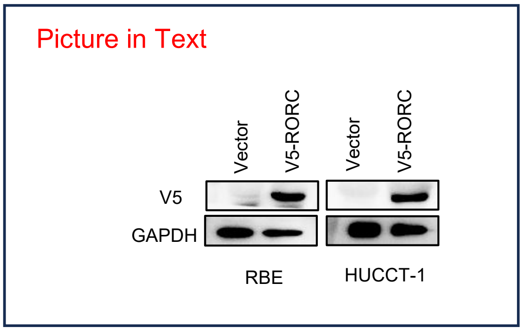

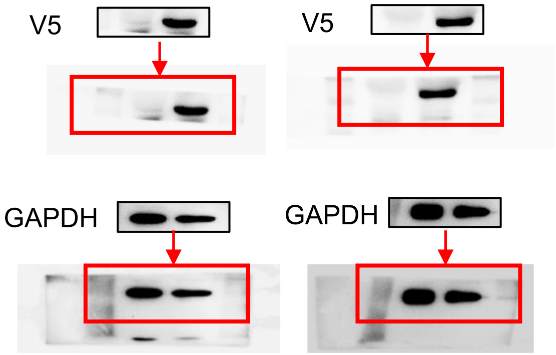

Supplement: Supplementary file 2 — Supplemental Material 2 [file 41420_2025_2844_MOESM2_ESM.pdf]
